# Cleavage of cFLIP restrains cell death during viral infection and tissue injury and favors tissue repair

**DOI:** 10.1126/sciadv.adg2829

**Published:** 2023-07-26

**Authors:** Kristel Martinez Lagunas, Deniz Pinar Savcigil, Matea Zrilic, Carlos Carvajal Fraile, Andrew Craxton, Emily Self, Iratxe Uranga-Murillo, Diego de Miguel, Maykel Arias, Sebastian Willenborg, Michael Piekarek, Marie Christine Albert, Kalvin Nugraha, Ina Lisewski, Erika Janakova, Natalia Igual, Wulf Tonnus, Ximena Hildebrandt, Mohammed Ibrahim, Marlies Ballegeer, Xavier Saelens, Andrew Kueh, Pascal Meier, Andreas Linkermann, Julian Pardo, Sabine Eming, Henning Walczak, Marion MacFarlane, Nieves Peltzer, Alessandro Annibaldi

**Affiliations:** ^1^ Center for Molecular Medicine Cologne, University of Cologne, Robert-Koch Strasse 21, 50931, Cologne, Germany.; ^2^ MRC Toxicology Unit, University of Cambridge, Tennis Court Road, Cambridge, CB2 1QR, UK.; ^3^ Aragón Health Research Institute (IIS Aragón), Biomedical Research Centre of Aragón (CIBA), Zaragoza, Spain.; ^4^ Department of Microbiology, Radiology, Pediatry and Public Health, University of Zaragoza, Zaragoza, Spain.; ^5^ CIBER de Enfermedades Infecciosas, Instituto de Salud Carlos III, Madrid, Spain.; ^6^ Department of Dermatology, University of Cologne, 50937 Cologne, Germany.; ^7^ Cologne Excellence Cluster on Cellular Stress Responses in Aging-Associated Diseases (CECAD), University of Cologne, 50931 Cologne, Germany.; ^8^ Institute of Biochemistry I, Medical Faculty, University of Cologne, 50931 Cologne, Germany.; ^9^ Division of Nephrology, Department of Internal Medicine 3, University Hospital Carl Gustav Carus at the Technische Universität Dresden, Dresden, Germany.; ^10^ Biotechnology Center, Technische Universität Dresden, Dresden, Germany.; ^11^ Department of Translational Genomics, University of Cologne, Weyertal 115b, 50931 Köln, Germany.; ^12^ VIB-UGent Center for Medical Biotechnology, VIB, B-9052 Ghent, Belgium.; ^13^ Department of Biochemistry and Microbiology, Ghent University, B-9000 Ghent, Belgium.; ^14^ Walter and Eliza Hall Institute of Medical Research, Parkville, VIC, Australia.; ^15^ Department of Medical Biology, The University of Melbourne, Parkville, VIC 3010, Australia.; ^16^ The Breast Cancer Now Toby Robins Research Centre, The Institute of Cancer Research, London, UK.; ^17^ Division of Nephrology, Department of Medicine, Albert Einstein College of Medicine, Bronx, NY, USA.; ^18^ Institute of Zoology, Developmental Biology Unit, University of Cologne, 50674 Cologne, Germany.; ^19^ Centre for Cell Death, Cancer, and Inflammation (CCCI), UCL Cancer Institute, University College, London WC1E 6BT, UK.

## Abstract

Cell death coordinates repair programs following pathogen attack and tissue injury. However, aberrant cell death can interfere with such programs and cause organ failure. Cellular FLICE-like inhibitory protein (cFLIP) is a crucial regulator of cell death and a substrate of Caspase-8. However, the physiological role of cFLIP cleavage by Caspase-8 remains elusive. Here, we found an essential role for cFLIP cleavage in restraining cell death in different pathophysiological scenarios. Mice expressing a cleavage-resistant cFLIP mutant, *Cflip^D377A^*, exhibited increased sensitivity to severe acute respiratory syndrome coronavirus (SARS-CoV)–induced lethality, impaired skin wound healing, and increased tissue damage caused by *Sharpin* deficiency. In vitro, abrogation of cFLIP cleavage sensitizes cells to tumor necrosis factor(TNF)–induced necroptosis and apoptosis by favoring complex-II formation. Mechanistically, the cell death–sensitizing effect of the D377A mutation depends on glutamine-469. These results reveal a crucial role for cFLIP cleavage in controlling the amplitude of cell death responses occurring upon tissue stress to ensure the execution of repair programs.

## Teaser

cFLIP cleavage acts as a molecular brake on cell death and is essential for the ability of tissues to overcome damage.

## INTRODUCTION

Cell death is a fundamental biological process that ensures tissue homeostasis and orchestrates tissue remodeling following injury or infection. However, if on the one hand abrogation of cell death responses can prevent the activation of repair programs, on the other hand exacerbated cell death can lead to tissue failure (*1*–*3*). Therefore, the ability of tissues to control the extent of cell death under stress conditions is of fundamental importance for the activation of optimal repair programs. Tumor necrosis factor (TNF) is a proinflammatory cytokine that is produced in response to a large variety of stressors, including viral infection and injury, and it can initiate repair processes by inducing the expression of proinflammatory genes or by triggering cell death (*1*, *4*, *5*). The mechanisms regulating the decision between these two outcomes are of fundamental importance for the maintenance of tissue homeostasis and for the capacity of tissues to overcome damage (*6*, *7*).

Binding of TNF to TNF receptor 1 (TNFR1) results in the formation of two spatially and temporally distinct complexes (*8*). A membrane-bound complex, called complex-I, is assembled on the intracellular death domain (DD)–containing portion of TNFR1. It is composed of adaptor proteins, such as TNFR1-associated death domain protein (TRADD) and TRAF2 (TNFR-associated factor 2); kinases, such as RIPK1 (receptor-interacting protein kinase 1), IKKα and IKKβ [inhibitor of nuclear factor κB (NF-κB) kinase subunits α and β], TAK1 (transforming growth factor β–activated kinase 1), TANK-binding kinase 1 (TBK1), and IKKε; and E3 ligases, such as cellular inhibitor of apoptosis protein 1 and 2 (cIAP1/2) and linear ubiquitin chain assembly complex (LUBAC) (*9*–*14*). The concerted action of phosphorylation and ubiquitination events ensures the correct assembly and stability of the complex, leading to the activation of genes required to mount an inflammatory response (*1*, *15*, *16*). Any perturbation of phosphorylation or ubiquitination processes leads to the formation of a secondary, cytoplasmic complex, referred to as complex-II (*1*, *14*, *16*–*21*). This complex consists of Fas associated via death domain (FADD), RIPK1, cellular FLICE-like inhibitory protein (cFLIP_L_), Caspase-8 and, depending on the cell types, RIPK3 (*18*, *22*). Complex-II has cytotoxic activity and can trigger Caspase-8–mediated apoptosis and RIPK1/RIPK3/mixed lineage kinase domain like pseudokinase (MLKL)–mediated necroptosis (*23*). In immune cells, Caspase-8 activation can result in gasdermin D cleavage with the consequent induction of pyroptosis (*24*). Apart from TNFR1, other immune receptors including Toll-like receptor 3 (TLR3), TLR4, and type I and type II interferon (IFN) receptors have the potential to induce a complex-II–like cell death–inducing platform (*25*–*27*). The cFLIP_L_/Caspase-8 heterodimer acts as a molecular switch that controls the cell death outcomes of complex-II. While the cFLIP_L_/Caspase-8 heterodimer is required to suppress necroptosis, the Caspase-8/Caspase-8 homodimer formation is required for the activation of downstream Caspase-3 and Caspase-7 and the induction of apoptosis (*28*–*30*). Therefore, cFLIP_L_ (here referred to as cFLIP) represents both an activator and an inhibitor of Caspase-8, because it is needed for the ability of the latter to suppress necroptosis, but at the same time, it prevents Caspase-8 full activation and apoptosis (*28*). cFLIP is a catalytically inactive homolog of Caspase-8, composed of tandem death effector domains (DED) and a caspase-like domain formed of a large and a small subunit (*31*). Apart from being the most direct, nonredundant regulator of Caspase-8 activity, cFLIP is also a Caspase-8 substrate. Caspase-8 can cleave cFLIP at aspartic acid-367 (D376) (D377 in mouse) (*32*). Different studies have addressed the function of the cleavage at D376 mainly using cell-free systems and hybrid protein expression (*31*, *33*, *34*). A recent report showed that mice bearing a noncleavable cFLIP mutant, where D371 and D377 were mutated to alanine, were born at the expected Mendelian ratio, and thymocytes derived from these double-mutant mice were more resistant to FasL-induced cell death than their wild-type (WT) counterparts (*35*). However, the biological role of the proteolytic event on cFLIP remains to be elucidated.

Here, we characterized the dynamics and function of cFLIP cleavage at D377 both in vivo and in vitro by generating a mutant mouse bearing a point mutation that abrogates the cleavage at D377 (*Cflip^D377A^* mice). The D377A mutation renders mice more sensitive to the lethal effect of severe acute respiratory syndrome coronavirus (SARS-CoV) infection, exacerbates the phenotype of the *Sharpin^cpdm^* mice, and impairs skin wound healing. Cells derived from the *Cflip^D377A^* mice are substantiallyt more sensitive to TNF-induced necroptosis and apoptosis and exhibit increased complex-II formation. At the mechanistic level, we observed that glutamine-469 (Q469), a residue important for cFLIP heterodimerization with Caspase-8, is responsible for the cell death–sensitizing effect of the cFLIP-D377A mutant. Therefore, we report on the precise biological function of cFLIP cleavage in keeping cell death responses in check during pathogen attack, tissue injury, and tissue damage, to favor remodeling and repair.

## RESULTS

### cFLIP cleavage by Caspase-8 limits TNF cytotoxicity

**Fig. 1.**
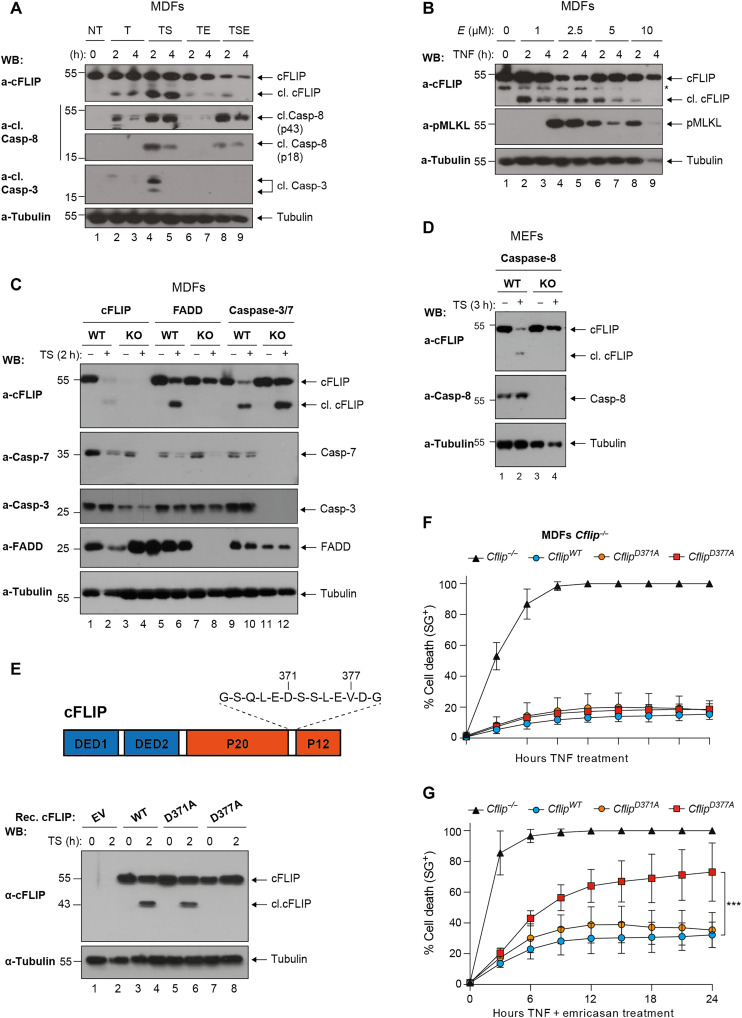
cFLIP is cleaved at D377 following TNFR1 activation. (**A**) WT MDFs were treated with TNF (T: 100 ng/ml), TNF and Smac mimetic (TS, S:250 nM), TNF and emricasan (TE, E:1 μM) and TNF, Smac mimetic and emricasan (TSE) for the indicated time points. Cell lysates were analyzed by immunoblotting for the indicated specific antibodies (*n* = 2). WB, Western blot. (**B**) WT MDFs were treated with TNF (100 ng/ml) and increasing concentration of emricasan for the indicated time points. Cell lysates were analyzed by immunoblotting with the indicated specific antibodies (*n* = 3). The asterisk (*) indicates a nonspecific band. (**C**) MDFs and (**D**) mouse embryonic fibroblasts (MEFs) of the indicated genotypes were treated with TNF (100 ng/ml) and Smac mimetics (250 nM) for 2 hours, and cell lysates were analyzed by immunoblotting with the indicated specific antibodies (*n* = 2). KO, knockout. (**E**) Cartoon depicting cFLIP domain composition and D371 and D377 (top). *Cflip^−/−^* MDFs were reconstituted either with empty virus or virus expressing WT or the indicated mutant versions of cFLIP and treated with TNF (100 ng/ml) and Smac mimetic (250 nM) for 2 hours. Cell lysates were analyzed by immunoblotting with the indicated specific antibodies (*n* = 2). (**F** and **G**) Cells as in (E) were treated with TNF (10 ng/ml) alone (F) or in combination with emricasan (G) (1 μM), and cell death was measured over time by calculating the percentage of Sytox Green–positive cells (SG^+^) (*n* = 4). Data are presented as means ± SD; **P < 0.01 (*n* = 4).

We first characterized the dynamics of cFLIP cleavage in TNF signaling in vitro. TNF treatment alone (T) or in combination with Smac mimetic (S) and/or emricasan (E) rapidly induced cleavage of cFLIP in mouse dermal fibroblasts (MDFs), as demonstrated by the appearance of the p43 fragment (Fig. 1A). Notably, cFLIP cleavage occurs in the absence of full Caspase-8 activation, as in the case of TNF-treatment alone, and despite the inhibition of Caspase-8 activity (Fig. 1, A and B). We next addressed the molecular requirements for cFLIP cleavage, and we found that FADD and Caspase-8, but not Caspase-3/7, are necessary for cFLIP to be cleaved (Fig. 1, C and D, and fig. S1, A to D). Because the co-deletion of Caspase-3 and Caspase-7 did not affect the cleavage event (Fig. 1C), we conclude that, upon TNF stimulation, the formation of a FADD/Caspase-8 complex is required for cFLIP cleavage and that Caspase-8 is most likely the only caspase able to cleave cFLIP in mouse cells. cFLIP bears an aspartic acid residue in close proximity to D377, the D371, which could potentially undergo Caspase-8–mediated cleavage (Fig. 1E) (*35*). To elucidate whether D371 can substitute for D377A, we reconstituted *Cflip^−/−^* MDFs with either WT cFLIP or mutant cFLIP bearing alanine substitutions in D371 or D377 (fig. S1E). We found that, whereas D371 is dispensable for cFLIP cleavage, D377 is the only cleavage site of cFLIP upon TNF signaling pathway activation (Fig. 1E and fig. S1F). Reexpression of the WT or mutant cFLIP variants could protect the *Cflip^−/−^* MDFs from TNF-induced cell death (Fig. 1F). Intriguingly, the cleavage-deficient D377A mutant cFLIP sensitized MDFs to TNF and emricasan (TE)–induced necroptosis (Fig. 1G). These findings indicate that cFLIP is cleaved by Caspase-8 following TNF signaling pathway stimulation at residue D377 and that this cleavage event has prosurvival functions.

### Noncleavable cFLIP sensitizes to TNF-induced necroptosis and apoptosis

**Fig. 2.**
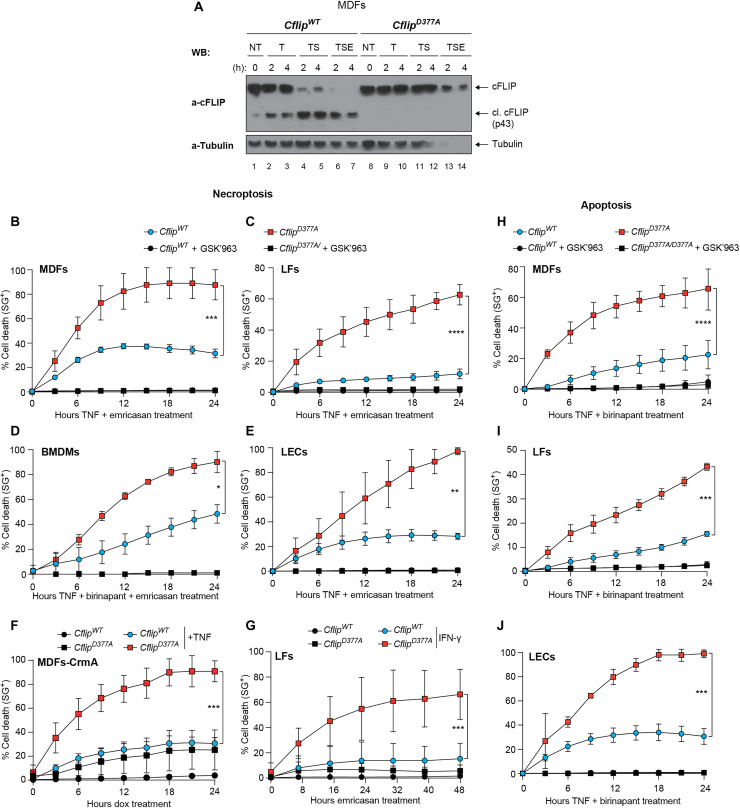
Cleavage-resistant cFLIP mutant sensitizes to TNF-induced necroptosis and apoptosis. (**A**) *Cflip^WT^* and *Cflip^D377A^* MDFs were treated as indicated [TNF (100 ng/ml), emricasan (1 μg/ml), and birinapant (250 nM)], and cell lysates were analyzed by immunoblotting using the indicated specific antibodies (*n* = 2). (**B**) WT and D377A mutant MDFs, (**C**) LFs, (**D**) BMDMs and (**E**) LECs were treated with TNF and emricasan (MDFs, LFs, and LECs) or TNF, birinapant, and emricasan (BMDMs) in the presence or not of the RIPK1-specific inhibitor GSK’963 (100 nM), and cell death was measured over time by calculating the percentage of Sytox Green–positive cells. For MDFs, TNF (1 ng/ml) and 1 μM emricasan (*n* = 6), for LFs, TNF (10 ng/ml) and 1 μM emricasan (*n* = 4) and for BMDMs and LECs, TNF (10 ng/ml), 250 nM birinapant, and 1 μM emricasan (*n* = 5 and *n* = 3, respectively). (**F**) C*flip^WT^* and *Cflip^D377A^* MDFs stably expressing a doxycycline-inducible HA-tagged CrmA construct were treated for 48 hours with doxycycline (1 μg/ml) and then treated or not with TNF (100 ng/ml). Cell death was measured as in (B) to (E) (*n* = 3). (**G**) WT and D377A mutant LFs were pretreated with IFN-γ for 18 hours and then subjected to emricasan (1 μm) treatment. Cell death was measured as in (B) to (E) (*n* = 3). (**H**) WT and D377A mutant MDFs, (**I**) LFs, and (**J**) LECs were treated with TNF and birinapant in the presence or not of the RIPK1-specific inhibitor GSK’963 (100 nM), and cell death was measured over time by calculating the percentage of Sytox Green–positive cells. For MDFs, LFs, and LECs, TNF (10 ng/ml) and 250 nM birinapant, *n* = 4. Data are presented as means ± SD; *P ≤ 0.05, **P ≤ 0.01, ***P ≤ 0.001, and ****P ≤ 0.0001.

To study the physiological function of cFLIP cleavage at D377, we generated cFLIP cleavage–resistant mice, referred to as *Cflip^D377A^* mice, where the D377 was mutated to alanine (fig. S2A). The *Cflip^D377A^* mutant mice were weaned at the expected Mendelian ratio and do not exhibit any overt phenotype (fig. S2, B to D). These mice had an overtly normal immune system (fig. S2E). MDFs isolated from WT and cFLIP mutant mice had comparable levels of cFLIP, and in the D377A mutant cells, cFLIP could not undergo proteolytic cleavage following TNFR1 pathway activation (Fig. 2A). Next, we tested the role of cFLIP cleavage in TNF-induced cell death. While *Cflip^D377A^* cells were not sensitive to TNF treatment alone (fig. S3A), MDFs, lung endothelial cells (LECs) and lung fibroblasts (LFs) were significantly more sensitive to TE- and bone marrow–derived macrophages (BMDMs) to TNF, emricasan, and Smac mimetic birinapant (TBE)–induced necroptosis, compared to their WT counterparts (Fig. 2, B to E). Consistent with the fact that RIPK1 kinase activity is required for necroptosis induction (*36*), the selective RIPK1 inhibitor GSK’963 suppressed TE- and TBE-mediated killing (Fig. 2, B to E). In addition, D377A mutant MDFs were also significantly more sensitive than WT MDFs to TNF treatment following expression of CrmA (*37*), a cowpox virus–encoded Caspase-8 inhibitor, via a doxycycline-induced construct (Fig. 2F and fig. S3B). Necroptosis can also be triggered by IFN-γ–induced Z-DNA-binding protein 1 (ZBP1) up-regulation, in combination with Caspase-8 inhibition (*25*). cFLIP mutant cells were also more sensitive than WT cells to IFN-γ and emricasan (IE)–induced necroptosis, in a TNF-independent manner (Fig. 2G and fig. S3C). cFLIP mutant cells were also more sensitive to TNF and birinapant (TB)– and TNF and cycloheximide (TC)–induced apoptosis (Fig. 2, H to J, and fig. S3D). TB-induced cell death was also RIPK1 kinase dependent, because the GSK’963 inhibitor could abolish cell death both in WT and mutant cells (Fig. 2, H to J). Last, the genotoxic drugs gemcitabine, paclitaxel, and doxorubicin, known to activate intrinsic apoptosis, killed WT and D377A mutant cells to the same extent (fig. S3, E to H). Together, these data indicate that cFLIP cleavage limits the ability of TNF to induce apoptosis and necroptosis in multiple cell types and also the ability of IFN-γ to trigger necroptosis, while it plays no role in cell death processes induced by genotoxins.

### The D377A mutation enhances complex-II formation, independently of NF-kB

**Fig. 3.**
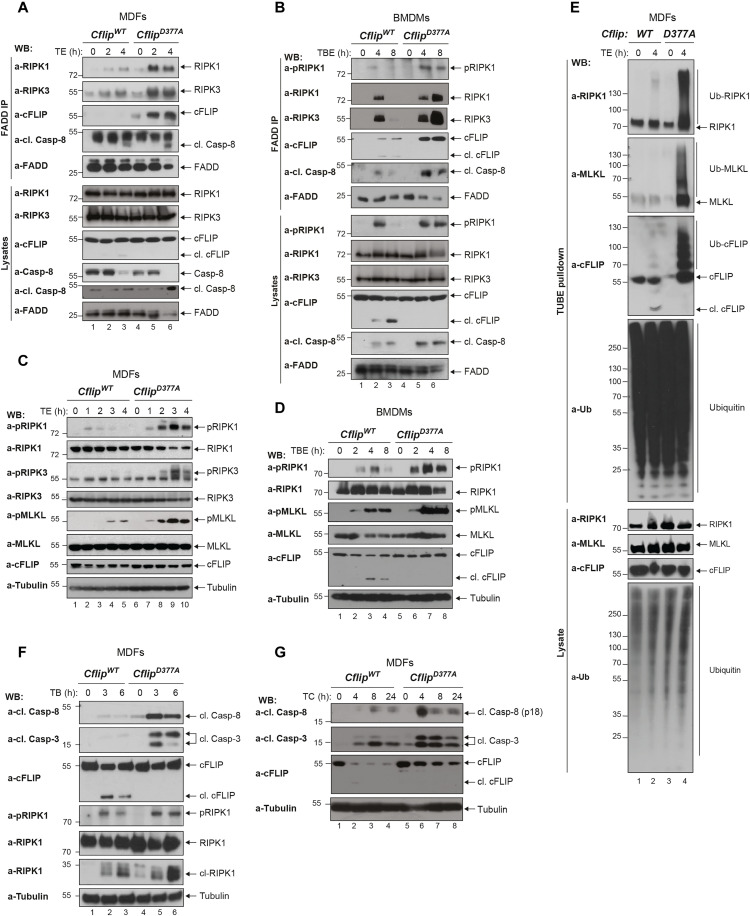
The D377A mutation enhances complex-II formation, independently of NF-κB. (**A**) WT and D377A mutant MDFs and (**B**) BMDMs were treated with TNF (1 ng/ml) and emricasan (1 μg/ml) and TNF (1 ng/ml), birinapant (250 nM), and emricasan (1 μg/ml), respectively, for the indicated time points. Cell lysates were subjected to immunoprecipitation using a FADD-specific antibody. Immunocomplexes and cellular lysates were then analyzed by immunoblotting using the indicated specific antibodies (*n* = 3). (**C**) MDFs and (**D**) BMDMs were treated as in (A) and (B) for the indicated time points. Cell lysates were analyzed by immunoblotting using the indicated specific antibodies (*n* = 2). (**E**) MDFs were treated with TNF (10 ng/ml) and emricasan (1 μg/ml), and cell lysates were subjected to TUBE pull-down, followed by immunoblotting analysis with the indicated specific antibodies (*n* = 3). (**F**) WT and D377A mutant MDFs were treated with TNF (10 ng/ml) and birinapant (250 nM) or (**G**) TNF (10 ng/ml) and cycloheximide (1 μg/ml) for the indicated time points, and cell lysates were immunoblotted using the indicated specific antibodies (*n* = 2).

TNF-induced necroptosis is driven by the formation of the RIPK1 kinase activity–dependent complex-II and the consequent phosphorylation of RIPK3 and MLKL (*23*, *36*). To ascertain how the abrogation of cFLIP cleavage sensitizes cells to necroptosis, we treated MDFs and BMDMs with TE or TBE, respectively, and immunoprecipitated complex-II via FADD. The subsequent immunoblotting analysis revealed increased association of RIPK1, RIPK3, Caspase-8, and cFLIP with FADD in the *Cflip^D377A^* cells (Fig. 3, A and B). In addition, we observed significantly higher levels of phosphorylated RIPK1, RIPK3, and MLKL in cFLIP mutant cells treated with TE, TBE, or IE, or expressing CrmA (Fig. 3, C and D, and fig. S4, A and B). In addition to phosphorylation, RIPK1 and MLKL also undergo ubiquitin modification that promotes their killing activity during necroptosis (*38*). Consistent with *Cflip^D377A^* cells being more sensitive to necroptosis, we observed markedly higher ubiquitination of RIPK1, MLKL, and cFLIP in the mutant cells compared to WT cells, upon TE treatment (Fig. 3E and fig. S4C). TNF-induced apoptosis is mediated by Caspase-8 activation, by means of autoproteolytic cleavage, which, in turn, activates the executioner caspases, Caspase-3 and Caspase-7 (*39*). Consistent with the observation that *Cflip^D377A^* cells are more sensitive to apoptosis, we observed increased cleaved Caspase-8 and cleaved Caspase-3 in cFLIP mutant cells treated with TB or TC (Fig. 3, F and G). Last, the enhanced sensitivity to cell death imparted by the cFLIP mutation is not because of defects in activation of NF-κB or MAPKs (mitogen-activated protein kinases), since these signaling pathways were not affected by the D377A mutation (fig. S4, D and E). Recent studies reported on the transcriptional role of Caspase-8 downstream of TLRs (*40*, *41*). Caspase-8 can cleave and inactivate N4BP1 to allow TLR-induced expression of proinflammatory cytokines (*40*). Therefore, we set up to understand whether cFLIP cleavage affects Caspase-8–mediated cytokine production. Western blot and reverse transcription quantitative polymerase chain reaction (qPCR) analysis of WT and *Cflip^D377A^* BMDMs treated with LPS, to stimulate TLR4, or Poly I:C, to stimulate TLR3, revealed a highly similar N4BP1 cleavage pattern (fig. S4F) and cytokine mRNA expression levels (TNF and CCL2) (fig. S4G) between the two genotypes. Collectively, these data demonstrate that the D377A mutation promotes complex-II formation and its ability to induce MLKL-dependent necroptosis and Caspase-3–dependent apoptosis, while it does not seem to affect the transcriptional programs activated by TNFR1 and TLRs.

### cFLIP cleavage arrests complex-II formation

**Fig. 4.**
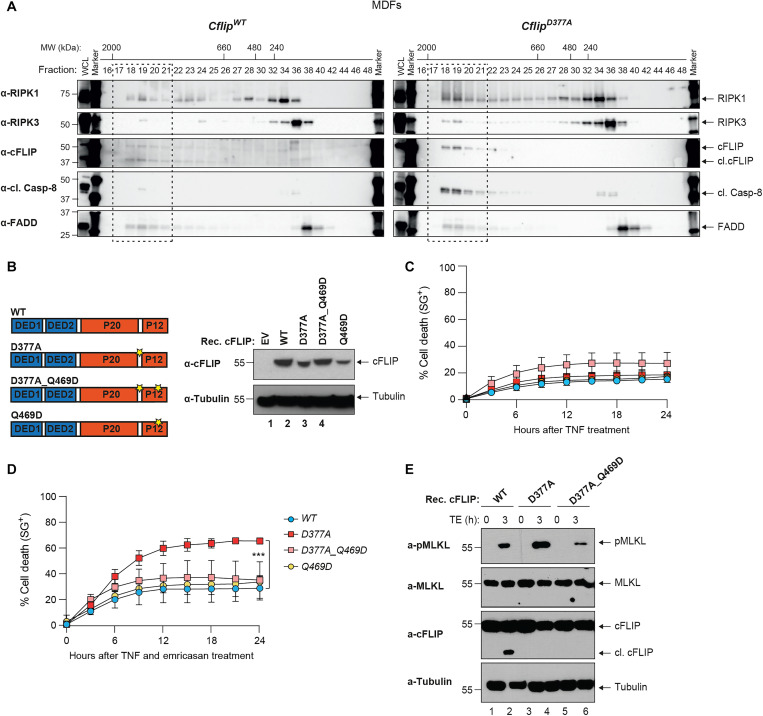
cFLIP cleavage counteracts complex-II formation. (**A**) *Cflip^WT^* and *Cflip^D377A^* MDFs were treated with TNF (10 ng/ml), Smac mimetic (250 nM), and emricasan (1 μg/ml) for 4 hours, and lysates were separated on a Superose 6 size exclusion column. Aliquots from each fraction were retained and analyzed by immunoblotting with the indicated specific antibodies. (**B**) Cartoon depicting cFLIP domain composition and position of the D377 and Q469 residues (left). Immunoblotting analysis of *Cflip^−/−^* MDFs reconstituted with the indicated cFLIP constructs via lentiviral infection or with an empty lentivirus (EV). cFLIP- and tubulin-specific antibodies were used (right). (**C** and **D**) *Cflip^−/−^* MDFs reconstituted as in (B) were treated with TNF (10 ng/ml) (C) or TNF (1 ng/ml) and emricasan (1 μg/ml) (D) and cell death was measured over time by calculating the percentage of Sytox Green–positive cells (*n* = 3). (**E**) MDFs *Cflip^−/−^* MDFs reconstituted as in (C) and treated as in (D) for 3 hours. Cell lysates were analyzed by immunoblotting with the indicated specific antibodies.

To mechanistically understand how cFLIP cleavage limits TNF-induced complex-II formation and cell death, we performed size exclusion chromatography. As expected, in WT cells treated with TBE, a small portion of RIPK1, RIPK3, cFLIP, and cleaved Caspase-8 co-eluted with FADD in fractions 17 to 21, which contain high molecular weight complexes of apparent molecular mass of ~2 MDa. Immunoprecipitation of FADD from pooled column fractions (17 to 21) further confirmed that these proteins are together in the same complex (fig. S5A). When gel filtration profiles of lysates from *Cflip^WT^* and *Cflip^D377A^* MDFs treated with TBE were compared, we observed increased abundance of RIPK1, RIPK3, cleaved Caspase-8, and cFLIP in fractions 17 to 21, corresponding to the high molecular weight complex, of *Cflip^D377A^* lysates (Fig. 4A). However, the D377A mutation did not further increase the mass of the high molecular weight complex, because in both *Cflip^WT^* and *Cflip^D377A^* MDFs complex-II components co-eluted in the same fractions (17 to 21). This corroborated the results obtained with FADD immunoprecipitation (Fig. 3, A and B) and indicated that cleavage of cFLIP at position D377 limits the extent of complex-II formation. We next reasoned that the p12 domain of cFLIP, which is C-terminal to the D377A, might be important for the assembly and stability of complex-II. This fragment contains a residue, Q469, which was reported to be involved in the process of heterodimerization with Caspase-8 (Fig. 4B) (*31*). Thus, cFLIP cleavage could regulate the formation of cFLIP/Caspase-8 heterodimers and, as a consequence, the assembly, stabilization and killing potential of complex-II. To interrogate this possibility, we reconstituted *Cflip^−/−^* MDFs with different cFLIP mutant constructs (Fig. 4B). Notably, the Q469D mutation did not impair the ability of cFLIP to protect cells from the cytotoxic effect of TNF alone (Fig. 4C). If our hypothesis were correct, then the Q469D mutation would abrogate the pro–cell death effects of the D377A mutation. While D377A-reconstituted cells were significantly more sensitive to TE-induced cell death than WT-reconstituted cells, the Q469D mutation reverted the sensitizing effect of the D377A mutation (Fig. 4D). The levels of TE-induced cell death in D377A/Q469D-reconstituted cells were comparable to those observed in WT- and Q469D-reconstituted cells. Consistent with this, phosphorylated MLKL levels in WT- and D377A/Q469D-reconstituted cells were reduced when compared to D377A-reconstituted cells (Fig. 4E). Together, these findings indicate that the cell death–sensitizing ability of D377A depends on Q469. This supports a model whereby cFLIP cleavage represents a molecular mechanism that controls the availability of a cFLIP/Caspase-8 interaction surface, the Q469, to limit the extent to which complex-II can form and mitigate cell death responses.

### Abrogation of cFLIP cleavage exacerbates the phenotype of *Sharpin* mutant mice

**Fig. 5.**
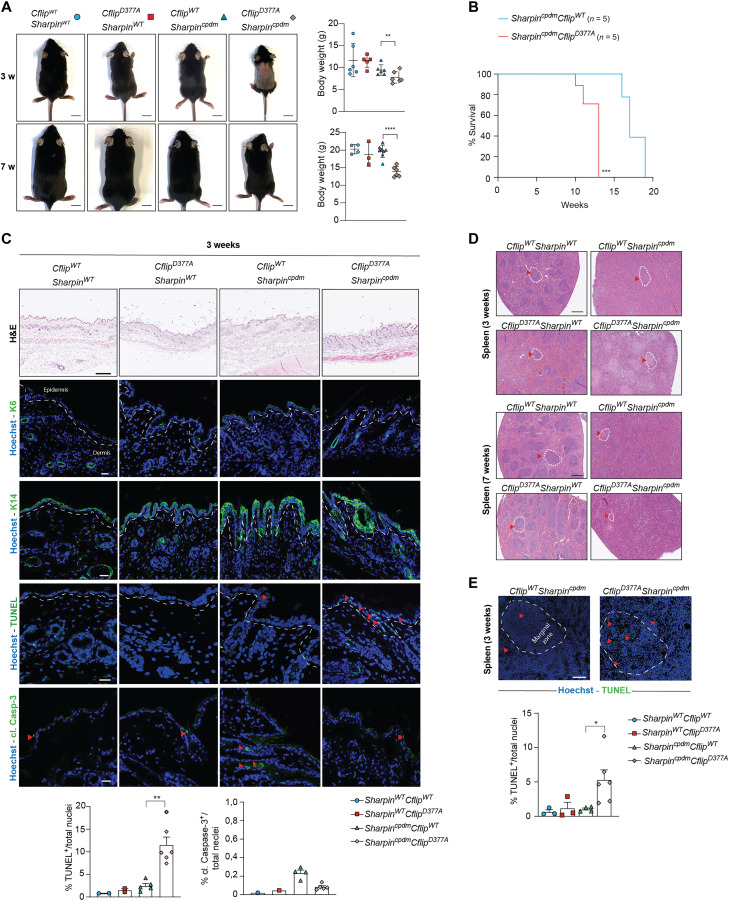
Abrogation of cFLIP cleavage exacerbates the phenotype of *Sharpin* mutant mice. (**A**) Pictures (left) and body weight (right) of 3- and 7-week-old *Cflip^WT^Sharpin^WT^*, *Cflip^D377A^Sharpin^WT^*, *Cflip^WT^Sharpin^cpdm^*, and *Cflip^D377A^Sharpin^cpdm^* mice. Each symbol corresponds to one mouse. Scale bars, 1 cm. Data are presented as means ± SD; **P < 0.01, ****P < 0.0001. (**B**) Kaplan-Meier survival curve of *Cflip^WT^Sharpin^cpdm^* and *Cflip^D377A^Sharpin^cpdm^* mice. The mice were euthanized when the termination criteria, based on the severity of dermatitis, were reached. ***P < 0.001. (**C**) Representative skin sections of 3-week-old mice of the indicated genotypes stained with H&E, K6, K14, cleaved Caspase-3, and TUNEL. Nuclei were stained with Hoechst. White dashed lines separate the epidermis from the dermis. Red arrowheads indicate TUNEL- and cleaved Caspase-3–positive cells. The graphics represent the percentage of TUNEL- and cleaved Caspase-3–positive cells over the total number of cells. Data are presented as means ± SD; **P < 0.01. Scale bars 20 μm. (**D**) Representative images of spleen sections of 3- and 7-week-old mice of the indicated genotypes [as in (A)] stained with H&E. Scale bars, 500 μm. Dotted circles and red arrowheads indicate marginal zones. (**E**) Representative pictures of TUNEL staining of spleen sections of 3-weeks-old mice of the indicted genotypes (top) and the relative quantification (bottom) expressed as percentage of TUNEL-positive cells over the total number of cells. Hoechst stains nuclei. Red arrowheads indicate TUNEL-positive cells. Data are presented as means ± SD; *P < 0.05. Scale bars, 100 μm.

Next, we sought to investigate in a genetic model of TNF-induced, cell death–mediated tissue damage whether the cFLIP cleavage had cell death–limiting functions. Sharpin deficiency in mice causes a TNF-mediated, cell death–dependent multiorgan inflammation, referred to as chronic proliferative dermatitis mice (cpdm), characterized by skin dermatitis, lung and liver inflammation, loss of marginal zones in the spleen, and loss of Peyer’s patches (PP) (*42*–*44*). We observed that skin extracts of 7-week-old *Sharpin^cpdm^* mice exhibited cleavage of cFLIP (fig. S6A). Because cFLIP cleavage at D377 limits TNF-induced complex-II–mediated cell death, we set up to investigate whether the loss of cFLIP cleavage would exacerbate the effects of *Sharpin* deletion in vivo. For this purpose, we generated *Cflip^D377A/D377A^Sharpin^cpdm/cpdm^* double-mutant mice (*Cflip^D377A^Sharpin^cpdm^*). *Cflip^D377A^Sharpin^cpdm^* mice were born at the expected Mendelian ratio, yet they were runted and displayed significantly lower body weight than *Sharpin^cpdm^* mice (Fig. 5A). Notably, the *Cflip^D377A^Sharpin^cpdm^* mice developed inflammatory skin lesions and had to be euthanized significantly earlier than the *Sharpin^cpdm^* mice because of these lesions (Fig. 5B). While 12-week-old *Sharpin^cpdm^* did not show any evident sign of dermatitis, *Cflip^D377A^Sharpin^cpdm^* mice exhibited damaged skin, increased epidermal thickness [hematoxylin and eosin (H&E)], and epidermal hyperplasia (K6), to an extent that was comparable to 18-week-old *Sharpin^cpdm^* mice with clear signs of dermatitis (Fig. 5B and fig. S6B). Consistently with an earlier onset of dermatitis, the skin of 3-week-old *Cflip^D377A^Sharpin^cpdm^* mice, although not visibly inflamed, had a significantly higher amount of terminal deoxynucleotidyl transferase–mediated deoxyuridine triphosphate nick end labeling (TUNEL)–positive cells than the skin of the *Sharpin^cpdm^* mice (Fig. 5C). Curiously, no increase in cleaved Caspase-3 was detected (Fig. 5C). TUNEL/K14 double staining showed that most dead cells in the skin of 3-week-old *Cflip^D377A^Sharpin^cpdm^* mice were keratinocytes (fig. S6C). These findings therefore support a model where the D377A mutation accelerates the *Sharpin* deficiency–mediated cell death of keratinocytes and the onset of dermatitis. Another feature of Sharpin-deficient mice is splenomegaly and loss of marginal zones. Although 7-week-old *Sharpin^cpdm^* mice exhibited splenomegaly, D377A mutation did not cause further spleen enlargement (fig. S6D). However, we observed a complete loss of marginal zones in *Cflip^D377A^Sharpin^cpdm^* mice, while *Sharpin^cpdm^* exhibited only minor spleen architecture alterations at 3 and 7 weeks of age (Fig. 5D). Consistent with this finding, we detected a significantly higher number of TUNEL-positive cells in the spleen of *Cflip^D377A^Sharpin^cpdm^* mice compared to *Sharpin^cpdm^* and control mice (Fig. 5E). Despite the loss of PPs (fig. S6E), histological analysis revealed that intestines of 7-week-old *Sharpin^cpdm^* mice were normal and largely comparable to those of control mice (fig. S6F). On the contrary, intestines of *Cflip^D377A^Sharpin^cpdm^* mice exhibited signs of damage, inflammation, and cell death, both in the small and large intestine, as shown by the histology score (fig. S6F), which correlates with a significant increase in the amount of TUNEL-positive cells (fig. S6G). Together, these findings indicate that the abrogation of cFLIP cleavage enhances the cell death–inducing potential of *Sharpin* deletion in vivo.

### cFLIP^D377A^ mice exhibit impaired skin wound healing

**Fig. 6.**
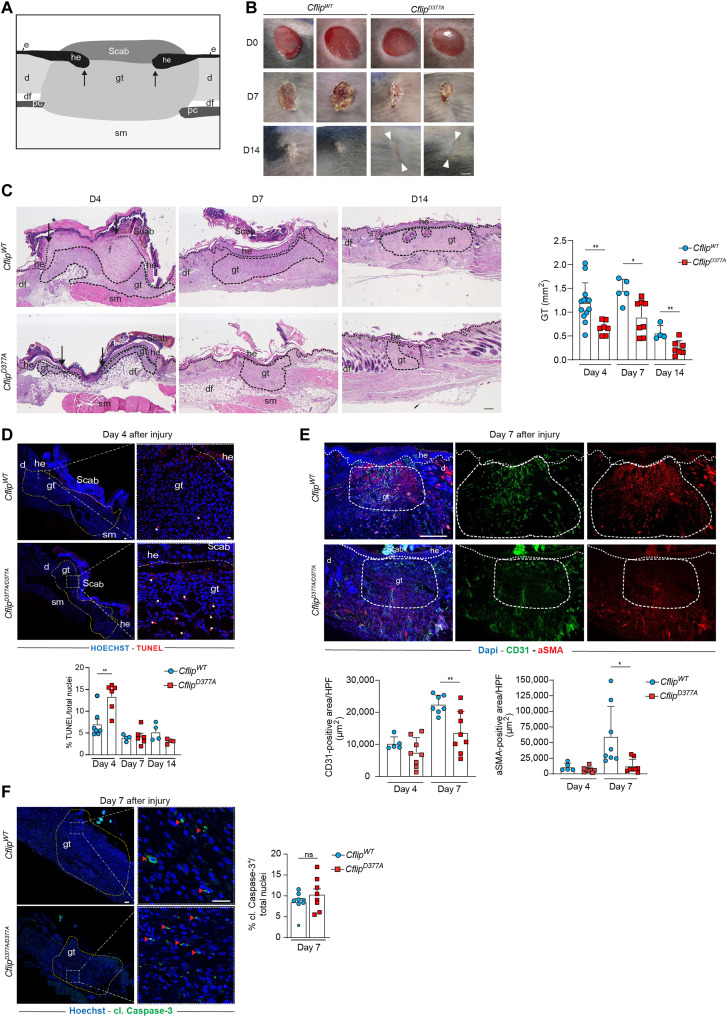
*Cflip^D377A^* mice exhibit impaired skin wound healing. (**A**) Cartoon depicting a skin wound at around 4 dpi (days post-injury), where the following skin areas are marked: d, dermis; df, dermal fat tissue; e, epidermis; gt, granulation tissue; he, hyperproliferative epithelium; pc, panniculus carnosus; sm, skeletal muscle. (**B**) Macroscopic pictures of wounds at 0, 7, and 14 dpi in *Cflip^WT^* and *Cflip^D377A^* mice. White arrowheads indicate scar tissue. Scale bar, 2 mm. (**C**) Representative H&E-stained wound sections (left) of *Cflip^WT^* (top) and *Cflip^D377A^* mice (bottom) at 4, 7, and 14 dpi. Scale bar, 200 μm. Arrows indicate the tips of the epithelial tongues. Quantitative analysis (right) of granulation tissue (gt) area of sections in (C) at 4, 7, and 14 dpi (*n* = 4 to 12 total wounds per genotype per dpi). (**D**) Representative TUNEL-stained wound sections (top) at 4 dpi of *Cflip^WT^* mice and *Cflip^D377A^* mice. Nuclei were stained with Hoechst (blue). Scale bars, 50 μm (left) and 20 μm (right). White asterisks indicate TUNEL-positive cells. Percentage of total TUNEL-positive cells (bottom) at 4, 7, and 14 dpi (*n* = 4 to 7 total wounds per genotype per dpi). (**E**) Representative CD31 (green) and α-SMA (red) immunostainings (top) at day 7 dpi of wound sections of *Cflip^WT^* and *Cflip^D377A^* mice. Scale bar, 200 μm. Percentage of total CD31- and α-SMA–positive area per HPF (high power field) in wound sections (bottom) at 4 and 7 dpi (*n* = 5 to 8 total wounds per genotype per dpi). (**F**) Representative cleaved Caspase-3–stained wound sections (left) at 7 dpi. Red arrowheads indicate cleaved Caspase-3–positive cells. Percentage of cleaved Caspase-3–positive cells (right) at 7 dpi (*n* = 4 to 7 total wounds per genotype per dpi). Data are presented as means ± SD; *P < 0.05 and **P < 0.01. ns, not significant.

To further validate the role of cFLIP cleavage in protecting tissues from cell death–induced damage, we used a model of mouse full-thickness excisional skin injury. Because Caspase-8 was reported to play a crucial role in the wound healing response (*45*) and it was very recently shown that wound healing is a TNF- and cell death–dependent process (*46*), we wondered whether Caspase-8–mediated cFLIP cleavage could contribute to wound closure. The wound closure is characterized by the formation of the granulation tissue in the damaged area that is composed of different cell types whose concerted action promotes wound closure (Fig. 6A) (*47*, *48*). Endothelial cells, myofibroblasts, and macrophages are the main components of the granulation tissue (*48*). Histomorphological analysis indicated a significant delay in the dynamics of wound closure in *Cflip^D377A^* mice as compared to WT mice, as shown by reduced scar formation (Fig. 6B) and reduced granulation tissue area at days 4, 7, and 14 after injury (Fig. 6C). Consistent with a reduction in the granulation tissue, *Cflip^D377A^* mice exhibited significantly higher levels of TUNEL-positive cells (Fig. 6D) at day 4 after injury, reduced wound vascularization (CD31 staining), and less myofibroblasts [α–smooth muscle actin (α-SMA) staining] at day 7 after injury (Fig. 6E). No difference in the percentage of cleaved Caspase-3–positive cells was detected (Fig. 6F). Thus, impairing cFLIP cleavage causes an increased cell death response that interferes with the wound healing response. We therefore conclude that cFLIP cleavage controls the extent of cell death occurring after wound injury to favor an optimal healing process.

### cFLIP^D377A^ mice are more sensitive to SARS-CoV–induced lethality

**Fig. 7.**
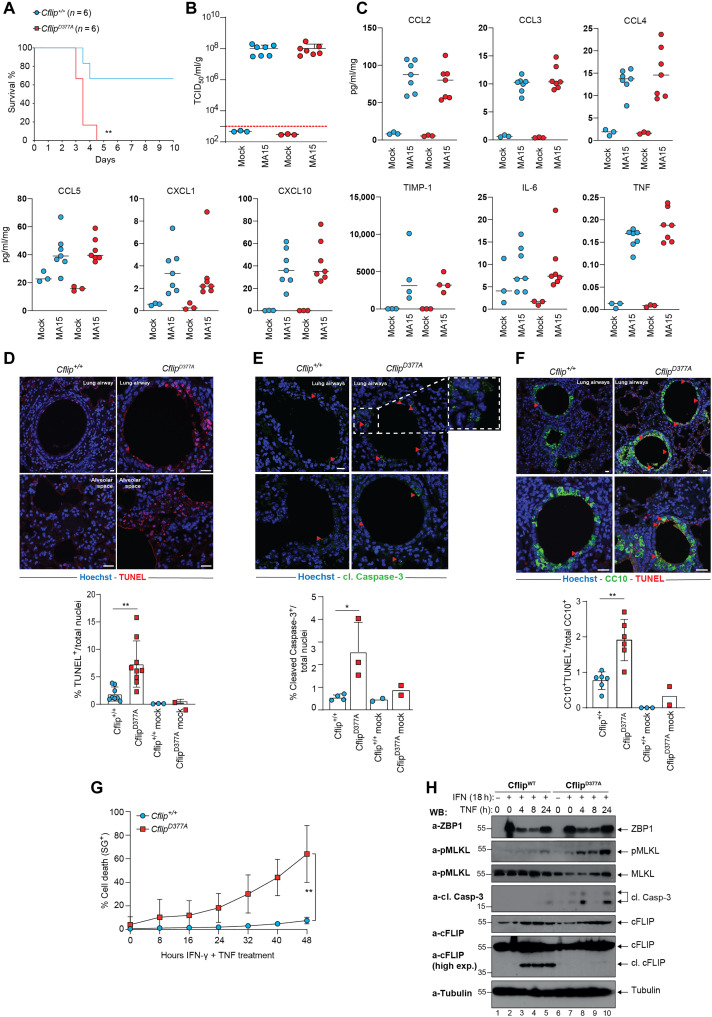
*Cflip^D377A^* mice are more sensitive to SARS-CoV–induced lethality. (**A**) Survival curve of *Cflip^WT^* and *Cflip^D377A^* mice infected with SARS-Cov MA15 virus (*n* = 6). **P < 0.01. Viral titer (**B**) and cytokine levels (**C**) from lungs of *Cflip^WT^* and *Cflip^D377A^* mice at 3 dpi (*n* = 3 for mock infection and *n* = 7 for MA15 infection). (**D**) Representative TUNEL-stained lung sections of *Cflip^WT^* mice and *Cflip^D377A^* mice 3 dpi (top). Nuclei were stained with Hoechst (blue). Scale bars, 10 μm (top left) and 20 μm (top right and bottom). Percentage of total TUNEL-positive cells (bottom) (*n* = 8 for *Cflip^WT^*-infected lungs, *n* = 10 *Cflip^D377A^*-infected lungs, and *n* = 2–3 for mock infection). Data are presented as mean ± SD; **P < 0.01. (**E**) Representative pictures of lung sections of *Cflip^WT^* and *Cflip^D377A^* mice at 3 dpi, stained with cleaved Caspase-3 (top). Nuclei were stained with Hoechst (blue). Scale bar, 10 μm. Red arrowheads indicate cleaved Caspase-3–positive cells. Percentage of total cleaved Caspase-3–positive cells (bottom). Data are presented as means ± SD; *P < 0.05. (**F**) Representative CC10 (green)/TUNEL (red) double-stained lung sections of *Cflip^WT^* mice and *Cflip^D377A^* mice at 3 dpi (top). Nuclei were stained with Hoechst (blue). Scale bars, 10 μm (top) and 20 μm (bottom). Red arrowheads indicate TUNEL-positive/CC10-positive cells. Percentage of total CC10/TUNEL double-positive cells (bottom). (*n* = 6 for *Cflip^WT^* infected lungs, *n* = 6 *Cflip^D377A^* infected lungs, and *n* = 2 to 3 for mock infection). Data are presented as means ± SD; **P < 0.01. (**G**) *Cflip^WT^* and *Cflip^D377A^* LFs were pretreated with IFN-γ (100 nM) for 18 hours and then treated with TNF (100 ng/ml) for additional 24 hours. Cell death was measured over time by calculating the percentage of Sytox Green–positive cells (*n* = 3). (**H**) Total lysates of LFs treated like in (G) for the indicated times points were immunoblotted with the indicated specific antibodies (*n* = 2).

Different types of viruses, including coronaviruses, can induce cell death by triggering Caspase-8–dependent apoptosis or ZBP1/RIPK3-dependent necroptosis, directly in the infected cells or as a consequence of the hyperinflammatory state created by the immune system (*49*–*51*). Our observation that the D377A mutation sensitizes cells to both apoptosis and necroptosis prompted us to investigate the biological role of cFLIP cleavage in a model of SARS-CoV infection. We therefore infected WT and *Cflip^D377A^* mice with a sublethal dose of a mouse-adapted strain of SARS-CoV-1 (MA15) (*52*), known to induce the production of TNF and other cytokines in the lung of infected mice (*53*, *54*). Notably, *Cflip^D377A^* mice were markedly more sensitive than WT mice to the lethal effect of the virus (Fig. 7A). However, we could not detect any difference in terms of viral titer between the two genotypes (Fig. 7B). We therefore reasoned that cFLIP cleavage is not involved in controlling viral replication. In addition, the levels of proinflammatory cytokines and chemokines between WT and mutant mice were largely comparable (Fig. 7C). When examining lung sections of infected mice, we detected a significantly higher number of TUNEL- and cleaved Caspase-3–positive cells in the lungs of infected *Cflip^D377A^* mice compared to infected WT mice, both in the bronchioles and alveoli (Fig. 7, D and E). In addition, costaining with TUNEL and CC10, a marker of club cells that are present in the respiratory bronchioles, revealed a higher number of TUNEL-positive club cells in *Cflip^D377A^* mice, indicating increased lung tissue damage (Fig. 7F). To explain the augmented cell death levels observed in the lungs of *Cflip^D377A^* mice, we considered the possibility that the cFLIP mutant lung cells exhibited higher sensitivity to the cytokines produced during the viral response. TNF and IFN-γ are among the main produced cytokines during SARS-CoV infection. Therefore, we pretreated WT and D377A mutant LFs with IFN-γ before TNF treatment and found that cFLIP mutant LFs were significantly more sensitive than their WT counterparts to IFN-γ/TNF-induced cell death (Fig. 7G). Immunoblotting analysis revealed that D377A mutant LFs underwent necroptosis and apoptosis, as shown by the higher levels of phosphorylated MLKL and cleaved Casapse-3 (Fig. 7H). This suggested that cFLIP cleavage can control the killing activity of a cytotoxic complex induced by TNF and IFN-γ. Together, these findings show that cFLIP cleavage represents a mechanism that protects mice from SARS-CoV–induced lethality by limiting the extent of cytokine-induced cell death, apoptosis, and potentially necroptosis occurring in the lungs.

## DISCUSSION

cFLIP is an essential, nonredundant cell death suppressor required to maintain tissue integrity (*55*–*60*). Its cell death inhibitory functions are due to the fact that it heterodimerizes with Caspase-8 and modulates its activity. Apart from being a modulator of Caspase-8 activity, cFLIP is also a Caspase-8 substrate, cleaved at position D377 (*32*). However, despite earlier studies on cFLIP proteolysis at D377 (*31*, *33*, *34*, *61*), the biological relevance of this cleavage event remained to be elucidated. Here, we generated mutant mice carrying a noncleavable cFLIP version, the *Cflip^D377A^*, which were born at the expected Mendelian ratio. It was previously shown that cFLIP is required for Caspase-8–mediated suppression of necroptosis. Our results indicate that the cleavage of cFLIP is dispensable for the ability of Caspase-8 to suppress necroptosis. When we analyzed different D377A mutant mouse cells, including BMDMs and LECs, we found that the abrogation of cFLIP cleavage sensitizes them to TNF-mediated apoptosis and necroptosis (induced by TNF and Smac mimetic as well as TNF and emricasan, respectively) by enhancing complex-II formation. The D377A mutation sensitizes cells not only to RIPK1-dependent cell death (apoptosis and necroptosis) but also to RIPK1-independent apoptosis (TNF and cycloheximide) and ZBP1-dependent necroptosis (IFN-γ and emricasan). Notably, in a previous study, a mutant mouse expressing noncleavable cFLIP was generated, where both D377 and the nearby D371 were mutated (*35*). Here, we show that the D377A mutation is sufficient to render cFLIP resistant to Caspase-8–mediated cleavage.

In vivo, the *Cflip^D377A^* mice were more susceptible to SARS-CoV–mediated lethality than WT mice, and this correlated with significantly higher levels of lung epithelial cell death, while no difference in viral titer or cytokine production was observed in lung extracts. Therefore, we envisage a scenario whereby the D377A mutation sensitizes lung cells to cell death induced by the overproduction of cytokines following lung infection, referred to as cytokine storm (*62*), which include TNF and IFN-γ. Therefore, the increased cell death observed in the lungs of mutant mice would not be the trigger but the consequence of the cytokine storm and the link between this last and the more severe lung pathology. Consistent with this scenario, we found that LFs from the *Cflip^D377A^* mouse are significantly more sensitive than WT cells to TNF/IFN-γ–induced cell death. Because IFN-γ induces the up-regulation of ZBP1 that can, in turn, trigger the assembly of a complex composed of RIPK1/RIPK3/Caspase-8/cFLIP (*63*), it is tempting to speculate that cFLIP cleavage can regulate the activity of this ZBP1-mediated cytotoxic complex. Recent reports showed that both Caspase-8–mediated apoptosis and MLKL-driven necroptosis contribute to disease severity following SARS-CoV infection (*41*, *50*, *51*, *62*). We detected both cleaved Caspase-3– and TUNEL-positive cells in the lungs of the infected *Cflip^D377A^* mice. In light of the fact that D377A mutant LFs treated with TNF/IFN-γ exhibited features of both apoptosis (cleaved Caspase-3) and necroptosis (phosphorylated MLKL) (Fig. 7, F to H), we hypothesize that noncleavable cFLIP can promote not only apoptosis but also necroptosis in the lungs of SARS-CoV–infected mice. In addition, *Cflip^D377A^* mice exhibited impaired wound healing following skin excision, as a consequence of increased cell death observed in the granulation tissue. Again, this would be consistent with a model whereby D377A mutation renders granulation tissue cells more sensitive to death induced by the cytokines, including TNF, produced during the wound healing processes likely by macrophages. To further provide understanding of the biological role of D377A mutation in vivo, we crossed the *Sharpin^cpdm^* mice, which carry an inactivating mutation on *Sharpin* that causes TNF-dependent cell death–mediated systemic inflammatory syndrome (*44*), with the *Cflip^D377A^* mouse. The resulting *Sharpin^cpdm^Cflip^D377A^* mice were runted; showed increased cell death levels in skin, spleen, and intestine in the first weeks after birth; and developed dermatitis significantly earlier than the *Sharpin^cpdm^* mice. At the mechanistic level, we were able to show that the cleavage of cFLIP controls the extent of complex-II formation by regulating cFLIP/Caspase-8 heterodimerization via Q469. Two pieces of evidence support this model: (i) In the D377A mutant cells stimulated with TNF, birinapant, and emricasan, there is a higher abundance of complex-II components co-eluting in the ~2 MDa gel filtration fractions. This suggests that the D377A mutation favors the assembly of complex-II. (ii) The Q469D mutation abrogates the cell death–sensitizing effects of the D377A mutation. This indicates that, in the absence of cFLIP cleavage, Q469 favors complex-II assembly and stability most probably by promoting cFLIP/Caspase-8 heterodimerization. Caspase-8 can, in turn, recruit more FADD molecules via DED-mediated interaction, which, in turn, can recruit more RIPK1 molecules via DD-mediated interaction. Therefore, the Caspase-8–mediated cleavage of cFLIP at position D377 represents a mechanism that counterbalances complex-II formation to keep its killing activity in check. Previous studies have reported conflicting results regarding the role of cFLIP cleavage on Caspase-8 activity, mainly using cell-free system assays containing recombinant or hybrid cFLIP and Caspase-8 (*31*, *33*, *34*). Such systems do not recapitulate the complexity of cell death–inducing platforms, such as TNF-induced complex-II, which need to be assembled to activate Caspase-8. Therefore, in light of our findings, we believe that cFLIP cleavage does not affect Caspase-8 activity per se, but rather acts indirectly by regulating the dynamics of a complex to which Caspase-8 is recruited and at which it is activated. Notably, Caspase-8 can also cleave other substrates in TNF-induced complex-II, such as RIPK1. While RIPK1 cleavage is a decisive event to prevent TNF from killing and the absence of cleavage results in mouse embryonic lethality and multiorgan inflammation in human (*35*, *64*, *65*), cFLIP cleavage is decisive in limiting TNF cytotoxicity once the TNF-mediated cell death arm is triggered. As a consequence, cFLIP cleavage becomes highly relevant under pathological conditions, where the extent of cell death have to be tightly regulated to prevent the detrimental effects of aberrant cell death. This highlights the different layers of regulation that complex-II is subjected to. The ability of tissues to overcome stress-induced damage lies also in their capacity to tightly control the amplitude of cell death responses, by modulating the formation and activity of cell death–inducing complexes. If in the absence of cell death tissue repair programs cannot be initiated, excessive cell death can lead to hyperinflammatory responses that are, in turn, detrimental for the tissue. Different types of insults, such as viral and bacterial infections or mechanical tissue damage, are known to activate a cFLIP/Caspase-8/RIPK1–containing cell death complex. The results presented here reveal that the cleavage of cFLIP is a required regulatory module in the intricated process that controls the activity of this cytotoxic complex, thereby ensuring optimal cell death responses and the consequent activation of tissue repair programs.

## MATERIALS AND METHODS

### Mice generation

The *Cflip^D377A^* mutant mice were generated by the MAGEC laboratory, Walter and Eliza Hall Institute of Medical Research (WEHI) on a C57BL/6J background. To generate these mice, Cas9 mRNA (20 ng/μl), single guide RNA (10 ng/μl; TTGATGGCCCATCTACCTCC), and oligo donor (40 ng/μl; GCCAAAGCTCTTTTTTATTCAGAACTATGAGTCGTTAGGTAGCCAGTTGGAAGATAGCAGTCTGGAGGTAGCTGGGCCATCAATAAAAAATGTGGACTCTAAGCCCCTGCAACCCAGACACTGCACAACTCA) were injected into the cytoplasm of fertilized one-cell stage embryos generated from WT C57BL/6J breeders. Twenty-four hours later, two-cell stage embryos were transferred into the uteri of pseudo-pregnant female mice. Viable offspring were genotyped by next-generation sequencing. Targeted animals were backcrossed twice to WT C57BL/6J to eliminate off-target mutations.

### Cell lines

Immortalized MDFs and LFs, Platinum-E, and human embryotic kidney 293T cells were cultured in Dulbecco’s modified Eagle’s medium (DMEM) supplemented with 10% fetal bovine serum (FBS), penicillin, and streptomycin under 10% CO_2_. LECs were seeded on 0.1% gelatine-coated wells and cultured in a 1:1 mixture of Endothelial Cell Growth Medium 2 (EGM2) (PromoCell) and fully supplemented DMEM (Merck) [containing 20% fetal calf serum, glucose (4 g/liter), 2 mM glutamine, 1% penicillin (100 U/ml)–streptomycin (100 μg/ml), sodium pyruvate 1% (1 mM), Hepes (20 mM), and 1% nonessential amino acids]. Vero E6 cells were supplied by J. Vergara from the Centro de Investigación en Sanidad Animal IRTA-CReSA (Barcelona, Spain). Vero E6 cells were grown in DMEM (Sigma-Aldrich) supplemented with 10% FBS (Sigma-Aldrich), 2 mM GlutaMAX (Gibco), penicillin (100 U/ml; Sigma-Aldrich), streptomycin (100 μg/ml; Sigma-Aldrich), amphotericin B (0.25 μg/ml; Sigma-Aldrich), 1% nonessential amino acids (Gibco), and 25 mM Hepes (Biowest).

### Isolation of primary cells and immortalization

MDFs were generated from the tail skin of 8- to 10-week-old mice. Once separated from the tail, the skin was minced with a scalpel and digested in 3 ml of trypsin (Sigma-Aldrich, T4049) for 1 hour at 37°C. Seven milliliters of DMEM was then added, the tube was manually shaken several times, and the digested tail was then filtered through a 100 μM cell strainer. Cells were washed once with phosphate-buffered saline (PBS) and seeded on a 6 cm plate. Two to 3 days later, they were infected with a SV40 Large T antigen expressing retrovirus for immortalization. LFs were generated from whole lungs of 8- to 10-week-old mice. Once separated from non-lung tissue, the lungs were cut in very small pieces and digested in 1.5 ml of collagenase type 2 (Worthington, 44N15307B) for 1 hour at 37°C and 500 rpm. One digested lung was seeded on two 15 cm dishes containing 20 ml of DMEM. After 2 days, the medium was changed and cells were grown until confluence. LFs were immortalized using an SV40 Large T antigen–encoding retrovirus.

LECs were isolated from the lungs of 8- to 12-week-old mice. Lungs were briefly washed in PBS, minced, and digested in 1 ml of 0.5% collagenase type II (Merck Millipore, C2-22) for 45 min at 37°C. The cell solution was then filtered through a 70 μm cell strainer and separated with magnetic beads (mouse CD31, Miltenyi Biotec) according to the manufacturer’s protocol. CD31^+^ endothelial cells were seeded on gelatin-coated wells and cultured in the endothelial cell medium mentioned above. After the first passage, cells were resorted using the same magnetic beads. Isolated primary endothelial cells were analyzed using the anti-CD31 antibody (Ab) to distinguish endothelial cells from other cell types. To generate BMDMs, bone marrow cells from tibia and femur of 2-month-old mice were seeded in noncoated petri dishes and cultured for 6 days in DMEM + 10% FBS + 20% (v/v) L929 mouse fibroblast–conditioned medium. Isolated cells were routinely tested negative for mycoplasma contamination by PCR.

### Reagents and Abs

The following reagents were used in this study: birinapant (MedChem Express, HY-16591), emricasan (Absource Diagnostics, S7775-0005), GSK’963 (SeleckChem, S8642), IFN-γ (PeproTech, 315-05-100), mouse TNF-α (Enzo Life Sciences, ALX-522-009-C050), and Sytox Green (Thermo Fisher Scientific, S7020). The following antibiotics were used: blasticidine (InvivoGen, ant-bl-1), hygromycin-B (InvivoGen, ant-hg-1/5), neomycin (InvivoGen, ant-gn-5), and puromycin (InvivoGen, ant-pr-1). The following Abs were used: Caspase-7 rabbit Ab (Cell Signaling Technology, 12827), CC10 (Santa Cruz Biotechnology, sc365992), CD31 (BD Pharmingen, 550274), cFLIP rabbit Ab (Cell Signaling Technology, 56343), cleaved Caspase-3 rabbit Ab (Cell Signaling Technology, 9661), cleaved Caspase-8 rabbit Ab (Cell Signaling Technology, 9429S), SMA-Cy3 conjugated (Sigma-Aldrich, C6198), FADD mouse Ab (Millipore, 05-486 /1F7), FADD rabbit Ab (Abcam, ab124812), MLKL rat Ab (EMD Millipore, MABC604), phospho-MLKL rabbit Ab (Abcam, ab196436), phospho-RIPK1 rabbit Ab (Cell Signaling Technology, 31122), Phospho-RIPK3 rabbit Ab (Cell Signaling Technology, 57220), RIPK1 mouse Ab (BD Bioscience, 610459), RIPK1 rabbit Ab (Cell Signaling Technology, 3493), RIPK3 rabbit Ab (ProSci Inc., 2283), tubulin mouse Ab (Thermo Fisher Scientific/Sigma-Aldrich, T9026/047M4789V), ubiquitin mouse Ab (Santa Cruz Biotechnology, SC-8017), and ZBP1 mouse Ab (AdipoGen, AG-20B-0010-C100).

### SARS-CoV infection

SARS-CoV-1 MA15 was provided by S. Zúñiga and L. Enjuanes (CNB-CSIC, Madrid, Spain). For the virus titration in lungs, previously weighed portions were homogenized in 500 μl of DMEM with a GentleMACS dissociator (Miltenyi), with centrifugation of 1500 rpm × 5 min, and the supernatant was taken. Virus titration was determined by median tissue culture infectious dose (TCID_50_)/ml assay performing serial dilutions and calculated using the Ramakrishan newly proposed method formula (*66*).

Mice were infected intranasally with 10^6^ TCID_50_/ml in a total volume of 40 μl of PBS after isoflurane anesthesia. Mice were weighed daily and reached humanitarian end point with a 25% weight loss. A clinical score was generated if needed, following different components: mouse appearance, level of consciousness, activity, response to stimuli, eye appearance, and frequency and quality of respiration, and mice reached humanitarian end point when the clinical score reached 21, when the respiratory characteristics were higher than 3, or when weight loss is greater than 25% of the initial weight. Animals were kept under standard conditions of temperature, humidity, and light at the Research Center on Encephalopathies and Emerging Diseases of the University of Zaragoza. Animal experimentation was approved by the Animal Experimentation Ethics Committee of the University of Zaragoza (number: PI44/20).

### Excisional punch injury

Mice were anesthetized by intraperitoneal injection of 100 mg/kg body weight of Ketavet (Pfizer) and 10 mg/kg body weight of Rompun 2% (Bayer). The back skin was shaved using an electric shaver and disinfected with 70% ethanol. Full-thickness punch biopsies were created on the back using a standard biopsy puncher (Stiefel). For histological analysis, wounds were excised at different times after injury and processed following as described in (*47*). The tissue was either fixed for 2 hours in Roti Histofix or embedded in O.C.T. compound (Thermo Fisher Scientific) and stored at −80°C.

### Retroviral production and infection

Replication-incompetent retroviral particles were generated in Platinum E cells. The cells were seeded in a 10 cm dish at 2,500,000 cells per dish in 10 ml of culturing medium. The following day, 1 ml of Opti-MEM I reduced serum medium containing 10 μg of plasmid of interest and 30 μl of polyethylenimine (1 mg/ml) was added to the medium and incubated overnight. The medium was changed after 18 hours of incubation. The supernatant containing retroviral particles was collected 2 days posttransfection. One day before the infection of target cells with retroviral particles, 80,000 cells per well were seed in six-well plate. On the day of infection, 3 ml of supernatant-containing retroviral particles and polybrene (1 μg/ml) were added to the respective wells of six-well plate and kept for 72 hours. To selectively expand the infected cells, a 7-day selection was performed using the respective antibiotics depending on the resistance cassette contained in the retroviral plasmid, i.e., puromycin (4 μg/ml), hygromycin (200 μg/ml), blasticidine (6 μg/ml), and neomycin (1 mg/ml).

### Cell culture, constructs, and transfection

The SV40 Large T antigen and all the mouse cFLIP WT and mutant coding sequences (D371A, D377A, D371A/D377A, and D377A/Q469D) were in the pBABE retroviral vectors, carrying puromycin, hygromycin, neomycin, or blasticidin resistance. *Cflip^−/−^* MDFs were generated from immortalized *Cflip^f/f^* MDFs infected with a Cre-expressing retrovirus, followed by antibiotic selection. All the reconstitutions of *Cflip^−/−^* MDFs were done by infection with retroviruses expressing the different cFLIP sequences, followed by antibiotic selection.

Mouse cFLIP WT was amplified from mouse cDNA and cloned into pBABE using Bam HI and Eco RI restriction enzymes. All the cFLIP mutants were generated by site-directed mutagenesis and clones into pBABE using Bam HI and Eco RI restriction enzymes.

### Complex-II purification

Cells were seeded in 10 cm dishes and treated as indicated using media containing mouse TNF (100 ng/ml) and emricasan (1 μM). Cells were lysed in 1% Triton X-100 lysis buffer [30 mM tris-HCl (pH 7.4), 120 mM NaCl, 2 mM EDTA, 2 mM KCl, 1% Triton X-100 supplemented with protease inhibitors, and 10 mM PR619] on ice. Cell lysates were rotated at 4°C for 20 min and then centrifuged at 4°C at 14,000 rpm for 15 min. Twenty microliters of protein G Sepharose (Sigma-Aldrich), previously blocked for 1 hour with lysis buffer containing 1% bovine serum albumin, was bound with FADD antibody (1.5 mg antibody/mg protein lysate) and was rotated with cleared protein lysates for 4 hours at 4°C. Three times washes in lysis buffer were performed, and immunocomplexes were eluted by boiling in 60 μl of 1× SDS Laemmli buffer.

### Tube assay

Cells were lysed in DISC lysis buffer [20 mM tris-HCl (pH 7.5), 150 mM NaCl, 2 mM EDTA, 1% Triton X-100, and 10% glycerol] supplemented with protease inhibitors, 1 mM dithiothreitol (DTT), PR619 (10 mM), and Glutathione S-Transferase (GST)–Tandem Ubiquitin Binding Entities (TUBE) (50 mg/ml; 50 mg of TUBE/mg protein lysate). Cell lysates were rotated at 4°C for 20 min and then clarified at 4°C at 14,000 rpm for 10 min. Twenty-five microliters of GST beads was added, and pull downs were performed overnight. Beads were washed three times in wash buffer [50 mM tris (pH 7.5), 150 mM NaCl, 0.1% Triton X-100, and 5% glycerol], and pulled down proteins eluted by boiling in 50 μl of 1× SDS Laemmli buffer.

### Immunostaining

Freshly isolated organs were fixed with 4% paraformaldehyde overnight, washed with PBS for 24 hours, and embedded in paraffin (lung, spleen, and skin) or cryopreserved (skin). Paraffin blocks and cryopreserved tissues were sectioned into 3 μm thick consecutive thick slices. After standard rehydration, a short antigen-retrieval with 1× sodium citrate (pH 6.0) (Sigma-Aldrich, C9999) was performed in a microwave for 5 min at 80% power. Tissue sections were then permeabilized with 0.2% (v/v) Triton X-100 in the Animal Free Blocker and Diluent (Vector Lab, SPS035) at room temperature (RT) for 10 min. Next, the sections were stained for the following antibodies [diluted in 0.2% (v/v) Triton X-100 in Animal Free Blocker and Diluent] overnight at 4°C: CC10 (1:50; Santa Cruz Biotechnology, sc365992), α-SMA–Cy3–conjugated (1:50; C6198, Sigma-Aldrich), and CD31 (1:50; 550274 BD Pharmingen). Note that, in CD31-stained cryosections, antigen retrieval was not performed. Primary antibodies were then visualized by secondary antibodies conjugated to Alexa Fluor 488 (1:200; Thermo Fisher Scientific, A-11008) diluted in 0.2% (v/v) Triton X-100 in Animal Free Blocker and Diluent containing 1:1000 Hoechst 33342 for nuclei staining at RT for 1 hour. For the detection of late PCD^+^ cells, the ApopTag Red In Situ Apoptosis Detection Kit (Merck S7165) was used. Briefly, sample sections were washed twice with PBS and treated with equilibration buffer for 10 s, incubated with the working strength terminal deoxynucleotidyl transferase enzyme for 1 hour at 37°C, followed by 10 min of stop buffer, and subsequent 30 min of rhodamine antibody solution at RT. Slides were quickly washed with PBS and mounted with ProLong Gold Antifade Mountant (Thermo Fisher Scientific, P36934). Last, images were acquired with a confocal fluorescence microscope (Stellaris 5 LIAchroic inverse).

### Intestine histological score

Formalin-fixed and paraffin-embedded intestinal Swiss rolls were sectioned (3 μm) and stained with H&E. Histological evaluation was performed using the scoring system as described in (*67*). Briefly, histopathology scores are composed of four parameters: epithelial hyperplasia, epithelial injury, tissue inflammation, and epithelial cell death. Histological subscores for each parameter: 0, absent; 1, mild; 2, moderate; and 3, severe. An “area factor” for the fraction of affected tissue was assigned and multiplied with the respective parameter score (1 = 0 to 25%; 2 = 25 to 50%; 3 = 50 to 75%; and 4 = 75 to 100%). Each area was scored individually and multiplied with the correlating area factor. Total histology score was calculated as a sum of all parameter scores multiplied with their area factors. Maximum score was 48. Evaluation was performed in a blinded fashion.

### Cell death analysis

Cells were seeded at 8000 cells (MDFs and LFs), 10,000 cells (LECs), and 50,000 cells (BMDMs) per well of a 96-well plate the day before the experiment. The following day, they were treated as indicated in the figure legends in the presence of 5 μM Sytox Green. Live uptake of Sytox Green by dead cells was monitored every hour over a period of 24 or 48 hours via an IncuCyte S3. The percentage of dead cells was calculated by using the Basic Analyzer of the IncuCyte 2020B software and the metric Sytox Object count per well normalized to the area confluence. The positive control (cells treated with TNF, Smac mimetic, and emricasan) was taken as 100% death, and with this, the percentage of cell death over time was calculated.

### Protein expression and purification

The pGEX-GST-TUBE construct was transformed into BL21 (DE3) cells and cultured in LB medium at 37°C. Protein expression was induced overnight at 18°C with 0.1 mM isopropyl-β-d-thiogalactopyranoside when OD_600_ (optical density at 600 nm) reached 0.6. Cells were centrifuged at 4500 rpm for 20 min, resuspended in lysis buffer (PBS + 300 mM NaCl and 1 mM DTT supplemented with protease inhibitors), and sonicated four times for 30 s at maximum amplitude. Lysates were then spun for 30 min, 4°C, 4500 rpm, and cleared lysates were added to GST beads O.N. in rotation at 4°C. Beads were then washed and GST-TUBE eluted using elution buffer [50 mM tris-HCl (pH 8.5), 150 mM NaCl, 1 mM DTT, and glutathione (6 mg/ml)] for two consecutive times. The elution product was dialyzed using a Slide-A-Lyzer cassette in TBS buffer [50 mM tris (pH 7.5) and 150 mM NaC]/1 mM DTT and stored at −80°C.

### Gel filtration

Cellular lysates were separated on a Superose 6 HR 10/30 size exclusion column and an AKTA purifier protein purification system (GE Healthcare), essentially as described previously (*68*). Aliquots from each fraction were retained for Western blotting, and fractions 12 to 16, 17 to 21, 22 to 26, 27 to 31, 32 to 36, 37 to 41, 42 to 46, and 47 to 51 were pooled and used for immunoprecipitation experiments.

### Reverse transcription qPCR

RNA samples were isolated using the commercially available RNA isolaton kit (Zymo Research) according to the manufacturer’s instructions. cDNA was synthesized using the MMLV reverse transcriptase (Promega) according to the manufacturer’s instruction. qPCR was performed using the ORA qPCR Green ROX L Mix (HighQu) according to the manufacturer’s instruction and a real-time PCR thermocycler (Bio-Rad). The amount of mRNA detected was normalized to actin mRNA values. Primer sequences are the following: actin forward: ATGGTGGGAATGGGTCAGAAGGAC, actin reverse: CATTGTAGAAGGTGTGGTGC, TNF forward: CATCTTCTCAAAATTCGAGTGACAA, TNF reverse: TGGGAGTAGACAAGGTACAACCC, CCL2 forward: CCACTCACCTGCTGCTACTCAT, and CCL2 reverse: TGGTGATCCTCTTGTAGCTCTCC.

### Statistical analysis

The number of independent experiments for each dataset is stipulated in the respective figure legends. Comparisons were performed with Student’s *t* test, repeated-measure analysis of variance (ANOVA), and log-rank Mantel-Cox test (Fig. 7A), whose values are represented in the figures as*P ≤ 0.05, **P ≤ 0.01, ***P ≤ 0.001, and ****P ≤ 0.0001 using Prism v.8.2 (GraphPad).
