# Supplementary material for: Cleavage of cFLIP restrains cell death during viral infection and tissue injury and favors tissue repair
Source: Sci Adv. 2023 Jul 26;9(30):eadg2829. doi: 10.1126/sciadv.adg2829 (PMC10371024; doi:10.1126/sciadv.adg2829)
Supplement: Supplementary file 1 — Figs. S1 to S6 [file sciadv.adg2829_sm.pdf]

Science Advances
AAAS

Supplementary Materials for

Cleavage of cFLIP restrains cell death during viral infection and tissue injury and favors tissue repair

Kristel Martinez Lagunas *et al.*

Corresponding author: Alessandro Annibaldi, a.annibaldi@uni-koeln.de

Sci. Adv. **9**, eadg2829 (2023)
DOI: 10.1126/sciadv.adg2829

This PDF file includes:

Figs. S1 to S6

Supplementary figure 1

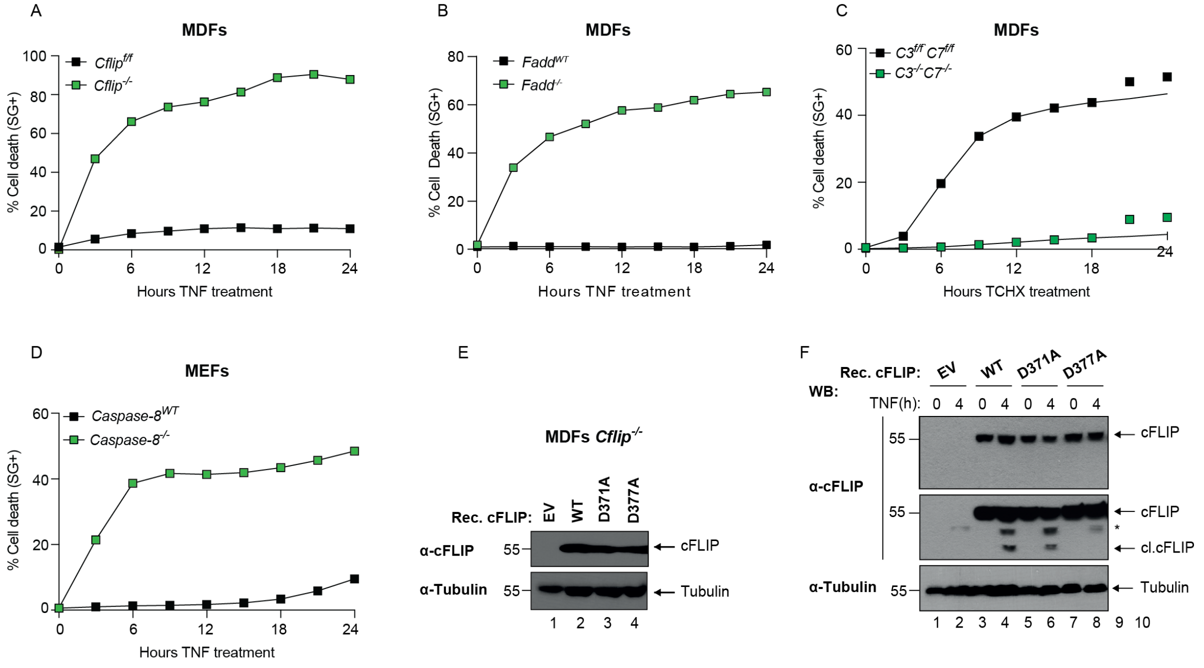

Supplementary Fig. 1. cFLIP is cleaved at Asp377 following TNFR1 activation. (A) *Cflip*$^{f/f}$ and *Cflip*$^{-/-}$ and (B) *Fadd*^{WT} and *Fadd*$^{-/-}$ were treated with TNF (100 ng/ml), (C) *Caspase3*$^{f/f}$*/Caspase7*$^{f/f}$ and *Caspase-3*$^{-/-}$*Caspase7*$^{-/-}$ MDFs were treated with TNF (100 ng/ml) and cycloheximide (1 µg/ml), (D) *Caspase-8*^{WT} and *Caspase-8*$^{-/-}$ MEFs were treated with TNF (100 ng/ml) and cell death was measured over time by calculating the percentage of Sytox Green positive cells. (E) Lysates of *Cflip*$^{-/-}$ MDFs, reconstituted as shown in the figure, were immunoblotted with the indicated specific antibodies (EV: empty vector). (F) *Cflip*$^{-/-}$ MDFs, reconstituted as in E were treated with TNF (10 ng/ml) and cell lysates were analyzed by immunoblotting with the indicated specific antibodies (n=2). The asterisk (*) indicates a non-specific band. (G) Cells as in E were treated with TNF (10 ng/ml) and cell death was measured over time by calculating the percentage of Sytox Green positive cells (graphic representative of two different experiments).

Supplementary figure 2

A

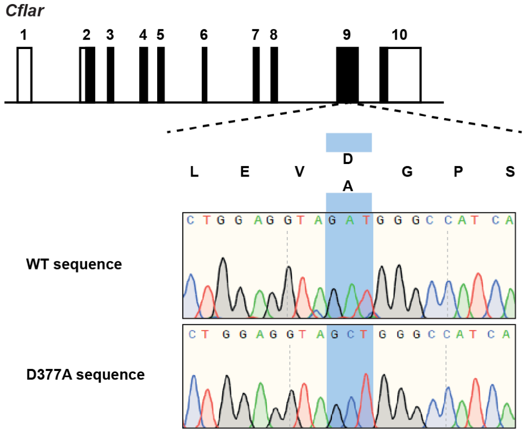

B

Genotype	# of mice	Observed (%)	Predicted (%)
Cflip$^{WT/WT}$	60	26,5	25
Cflip$^{WT/D377A}$	112	49,5	50
Cflip$^{D377A/D377A}$	54	24	25

C

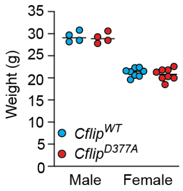

D

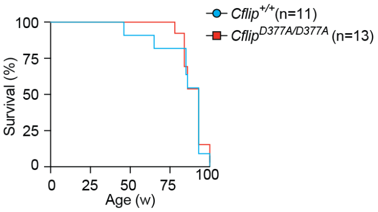

E

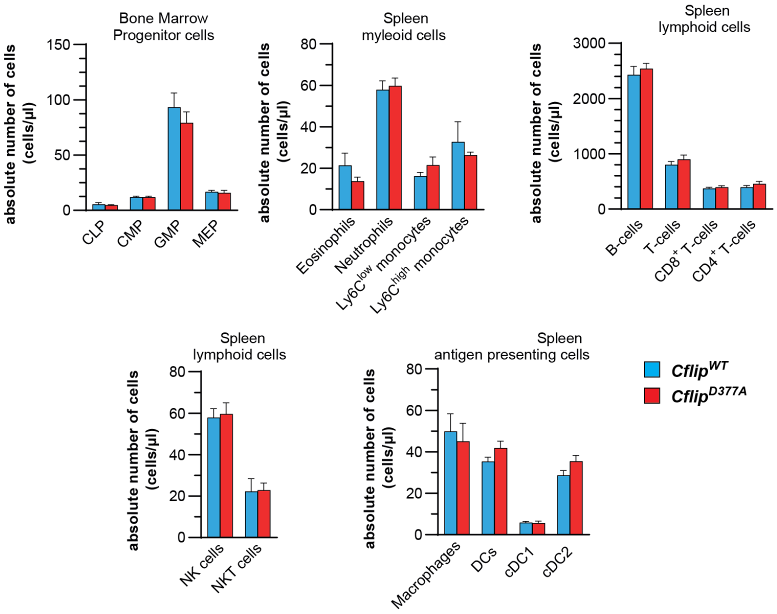

Supplementary Fig. 2. cFLIP is cleaved at Asp377 following TNFR1 activation. (A) Cartoon depicting the *Cflar* exons (upper part). DNA sequence alignment of the region bearing the D>A mutation (lower part). (B) Observed and expected numbers of mice of the indicated genotypes from $Cflip^{WT/D377A}$ intercrosses. (C) Weight of 12 weeks old $Cflip^{WT}$ and $Cflip^{D377A}$ homozygous mice. (D) Aging curves of $Cflip^{WT}$ and $Cflip^{D377A}$ homozygous mice. (E) FACS analysis of hematopoietic cells isolated from spleen and bone marrow of $Cflip^{WT}$ and $Cflip^{D377A}$ homozygous mice (n=4 mice per genotype).

Supplementary figure 3

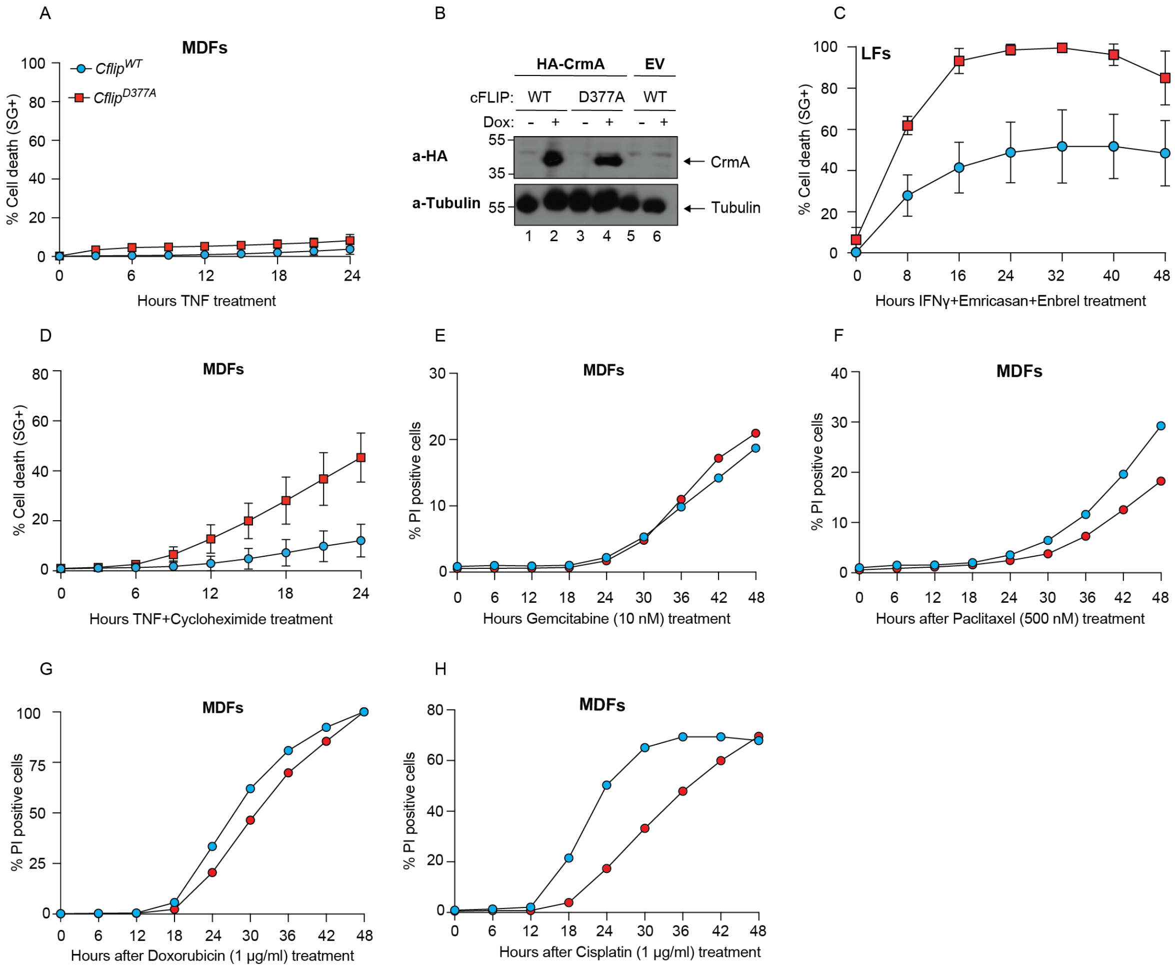

Supplementary Fig. 3. Cleavage resistant cFLIP mutant sensitizes to TNF-induced necroptosis and apoptosis. (A) $Cflip^{WT}$ and $Cflip^{D377A}$ MDFs were treated with TNF (100 ng/ml) and cell death was measured over time by calculating the percentage of Sytox Green positive cells. (B) MDFs cFLIP WT and D377A stably expressing a doxycycline-inducible HA-tagged CrmA construct and WT MDFs expressing an empty vector (EV) were treated for 72 hours with doxycycline (1µg/ml). Cell lysates were then analysed by immunoblotting using an anti-HA specific antibody. (C) LFs were pre-treated with Enbrel (50 µg/ml) and IFNγ before being subjected to emricasan (1 µM) treatment. Cell death was measured as in A. MDFs WT and D377A mutant were treated with TNF (10 ng/ml) and cycloheximide (1 µg/ml) (D), gemcitabine (10 nM) (E), paclitaxel (500 nM) (F), doxorubicin (1 µg/ml) (G) and cisplatin (1 µg/ml) (H), and cell death was measure as in A.

Supplementary figure 4

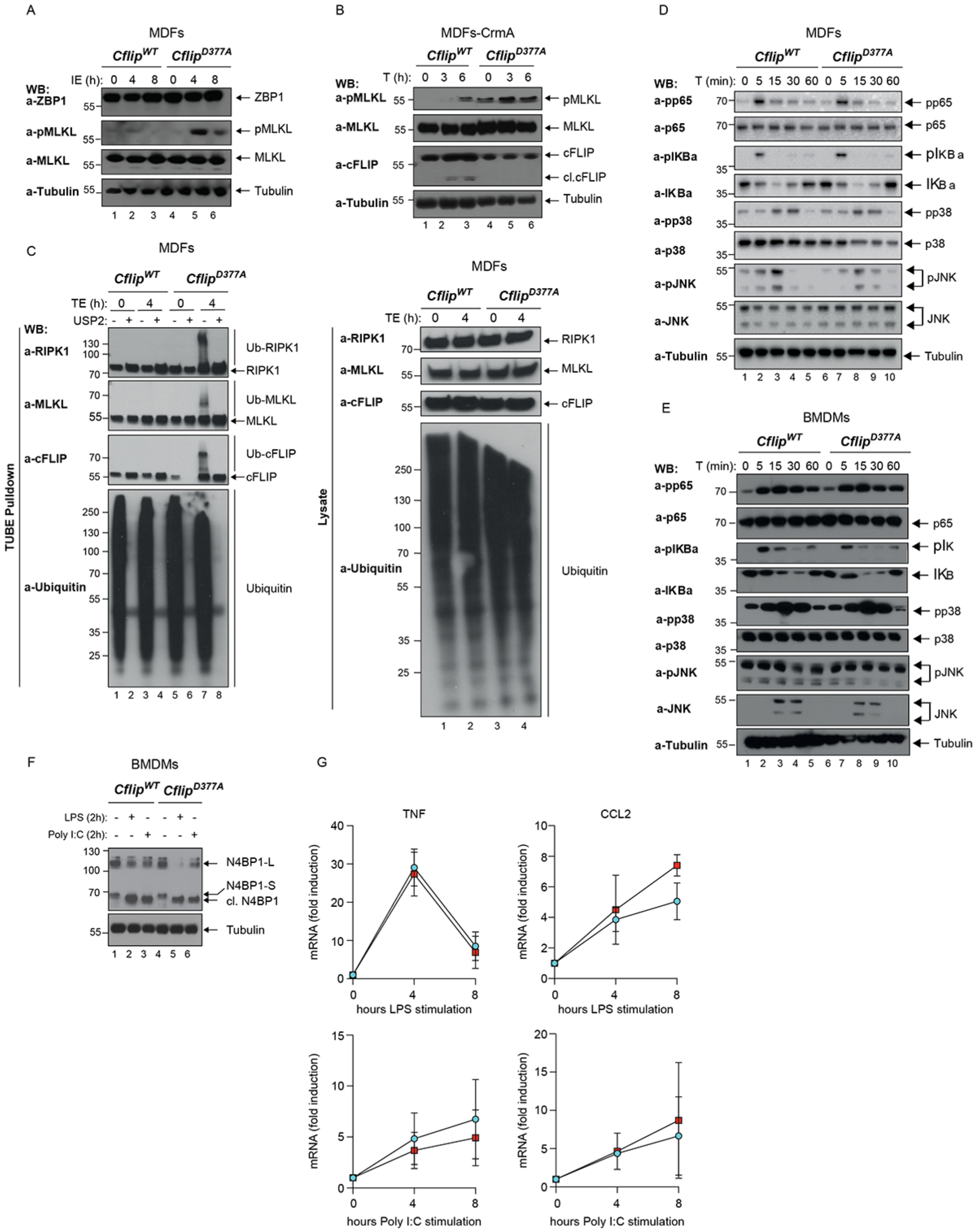

Supplementary Fig. 4. cFLIP cleavage limits complex-II formation, independent of NF-kB. (A) WT and D377A-mutant MDFs were treated with IFNγ (100 ng/ml) and emricasan (1 μM) for the indicated time points and protein extracts were analyzed by immunoblotting with the indicated specific antibodies (n=2). (B) WT and D377A mutant MDFs stably expressing a doxycycline (dox)-inducible construct encoding for CrmA were treated for 72 hours with dox and then with TNF (T) (100 ng/ml) for the indicated time points. protein extracts were analyzed by immunoblotting with the indicated specific antibodies (n=3). (C) $Cflip^{WT}$ and $Cflip^{D377A}$ MDFs were treated with TNF (1 ng/ml) and emricasan (1 μg/ml) for the indicated time points and protein lysates were subjected to TUBE pull-down. The pull-down fractions were left untreated or treated with the deubiquitinase USP2 and analysed by immunoblotting (n=3). (D) MDFs and (E) BMDMs were treated with TNF (100 ng/ml) for the indicated time points and protein extracts were analysed by immunoblotting with the indicated specific antibodies (n=2).

Supplementary figure 5

A

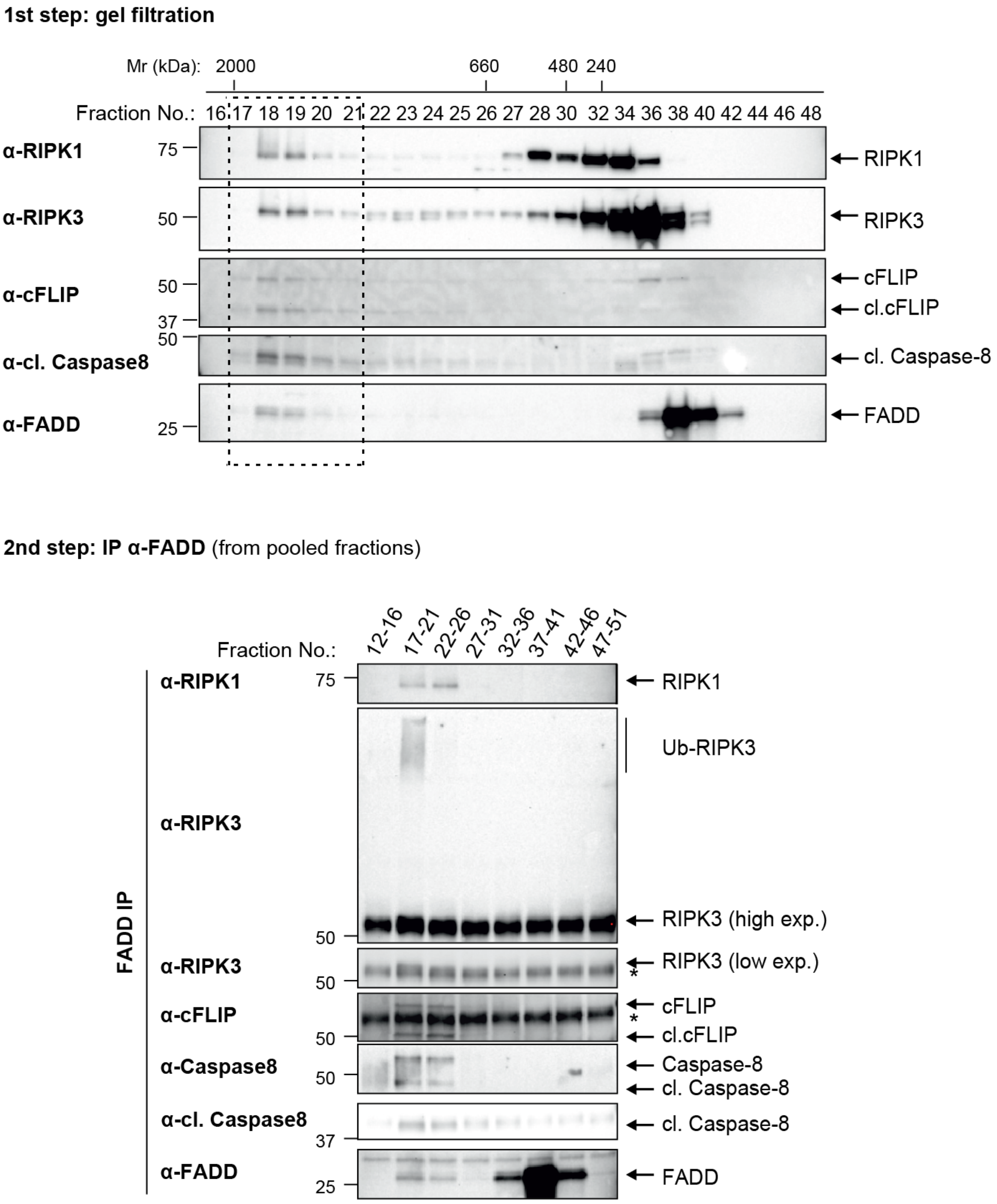

Supplementary Fig. 5. cFLIP cleavage counteracts complex-II formation. (A) WT MDFs were treated for 4 hours with TNF (10 ng/ml), Smac mimetic birinapant (250 nM) and emricasan (1 μg/ml) and lysates separated on a Superose 6 column. Aliquots from each fraction were retained and analyzed by immunoblotting with the indicated specific antibodies (1st step) or pooled as indicated and subjected to FADD immunoprecipitation (2nd step). Immunocomplexes were then analyzed by immunoblotting as indicated.

Supplementary figure 6

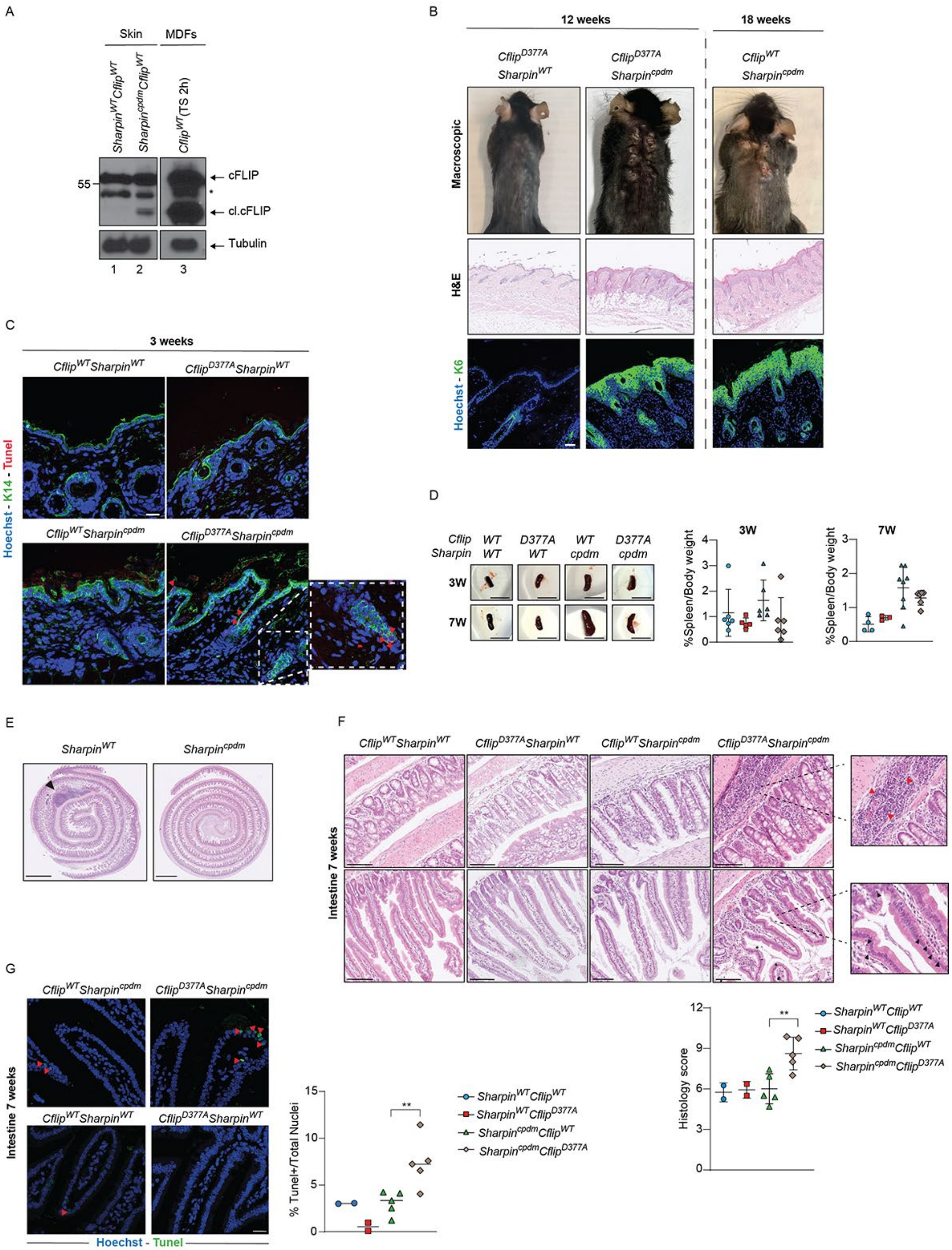

Supplementary Fig. 6. Abrogation of cFLIP cleavage exacerbates the phenotype of *Sharpin* mutant mice. (A) Skin lysates of 7 weeks old WT and $Sharpin^{cpdm}$ mice and lysate from WT MDFs treated for 2 hours with TS were immunoblotted for the indicated proteins. Right and left panel belong to the same film. (B) Representative pictures of mice and skin sections, stained with H&E and K6, of the indicated genotypes and age. (C) Representative pictures of skin sections, co-stained with K14 and TUNEL, of 3 weeks old mice of the indicated genotypes. (D) Spleen of 3 and 7 weeks old mice of the indicated genotypes (left), with the respective weight express as percentage of total body weight (right). (E) Small intestine sections of $Sharpin^{WT}$ and $Sharpin^{cdpm}$ mice. Arrowhead indicates a Peyer's patch. Scale bar 1 mm. (F) Large (top row) and small (lower row) intestine sections of 7 weeks old mice of the indicated genotypes with the histology score relative to the whole intestine. Each symbol represents one mouse. Data are presented as mean ± SD, **p<0,01. Scale bar 100µm. Red and dark arrowheads indicate areas of inflammation and dead cells, respectively. (G) TUNEL staining of small intestine sections of 7 weeks old mice of the indicated genotypes (left) with the relative quantification (right) expressed as percentage of TUNEL positive cells over the total number of cells. Scale bar 20 µm. Red arrowheads indicate TUNEL positive cells. Data are presented as mean ± SD, *p<0,05.
